# Supplementary material for: Geographic differences in gut microbiota composition impact susceptibility to enteric infection
Source: Cell Rep. 2021 Jul 27;36(4):109457. doi: 10.1016/j.celrep.2021.109457 (PMC8333197; doi:10.1016/j.celrep.2021.109457)
Supplement: Document S1. Figures S1–S7 and Tables S1 and S3 [file mmc1.pdf]

**Cell Reports, Volume *36***

## Supplemental information

# Geographic differences in gut microbiota composition impact susceptibility to enteric infection

**Ana Maria Porras, Qiaojuan Shi, Hao Zhou, Rowan Callahan, Gabriella Montenegro-Bethancourt, Noel Solomons, and Ilana Lauren Brito**

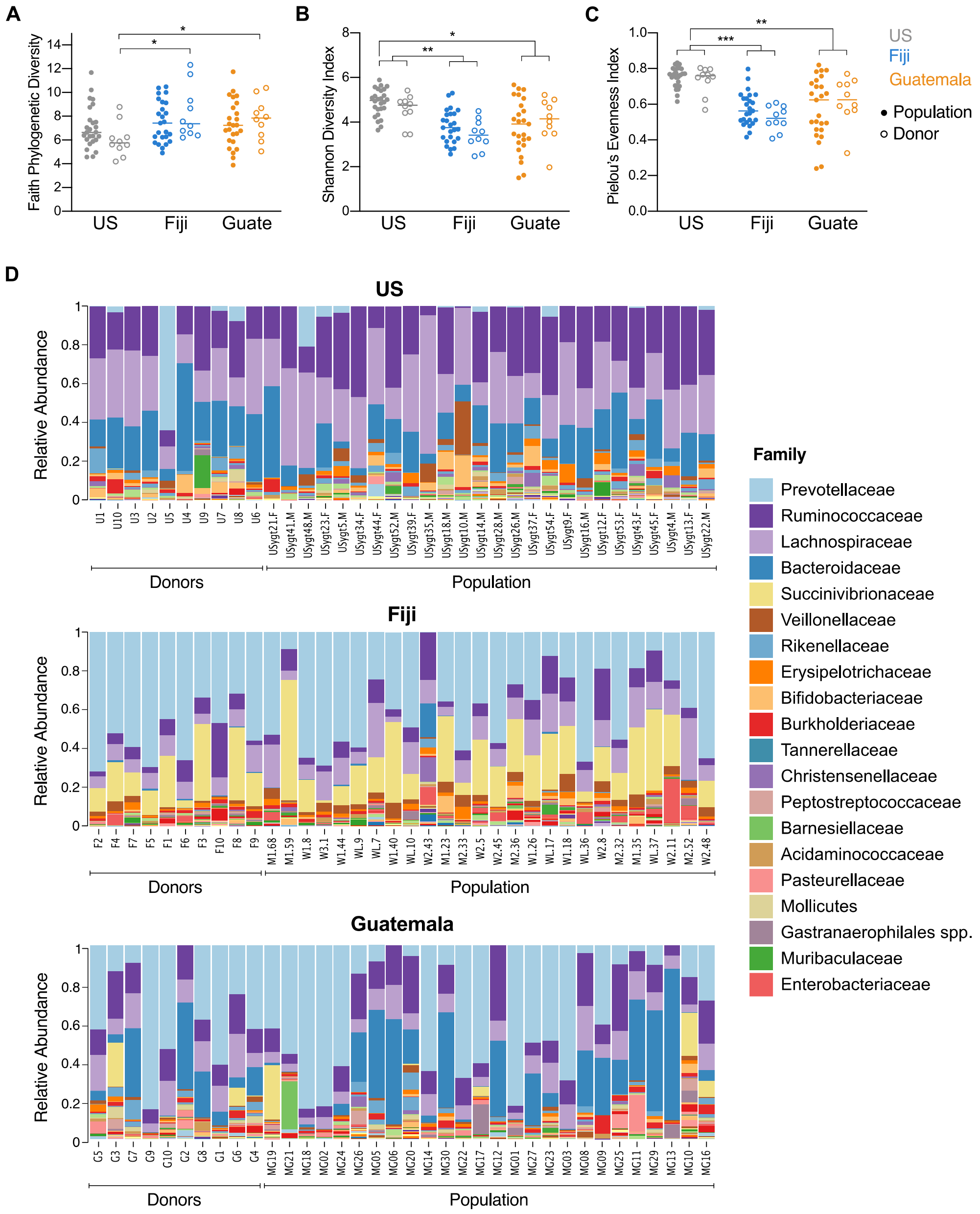


**Supplementary Figure 1. The microbiomes of selected human donors are representative of those of their corresponding populations.** Alpha diversity of the gut microbiomes of both donors and subjects from their populations were analyzed through (A) the Faith phylogenetic diversity index, (B) the Shannon diversity index and (C) Pielou's evenness index. (D) Abundance of bacteria genera in donors and their respective populations. Each bar represents an individual. Data are presented as mean values ± SD, with $*p<0.05$, $**p<0.01$, and $***p<0.001$ for comparisons shown (one-way ANOVA followed by Tukey's multiple comparison test). Related to Figure 1.

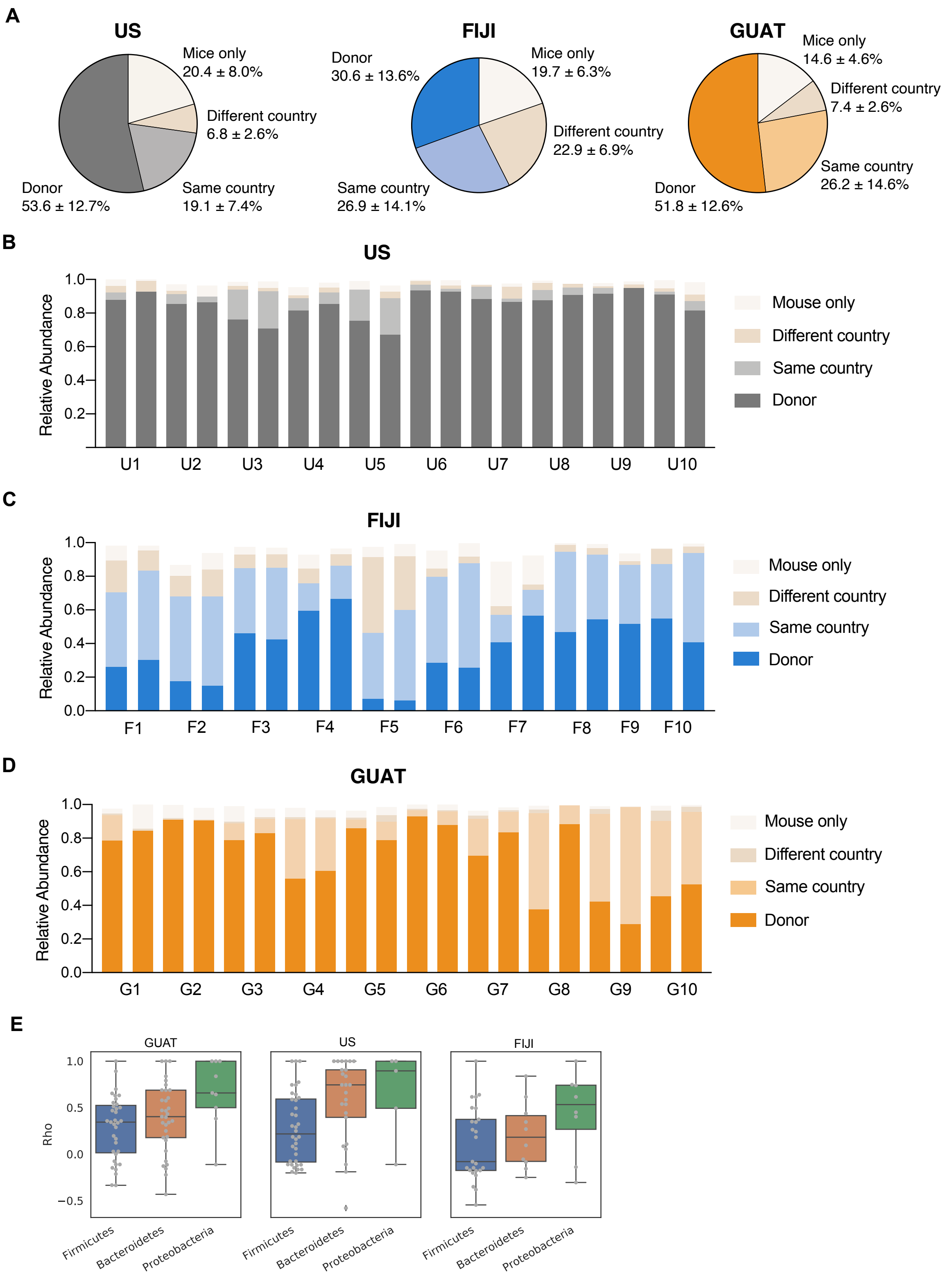


**Supplementary Figure 2. Human-associated microbes successfully colonize germ-free mice.** (A) Proportion of the SVs found in the microbiomes of the mice 14 days after microbiota transplantation that were originally found in the corresponding donor, in other donors of the same country, in donors of a different country, and only found in the

mice. We also calculated the relative abundances of the SVs found in the microbiomes of the (B) US, (C) FIJI, and (D) GUAT mice 14 days after microbiota transplantation that were originally found in: the corresponding donor, in other donors of the same country, in donors of a different country, or only found in the mice. (E) Boxplots showing colonization efficiency of ASVs transferred from the human microbiota to the mice at the phylum level 2 weeks after colonization. Transfer efficiency is represented by the Spearman correlation coefficients (rho). Related to Figure 1.

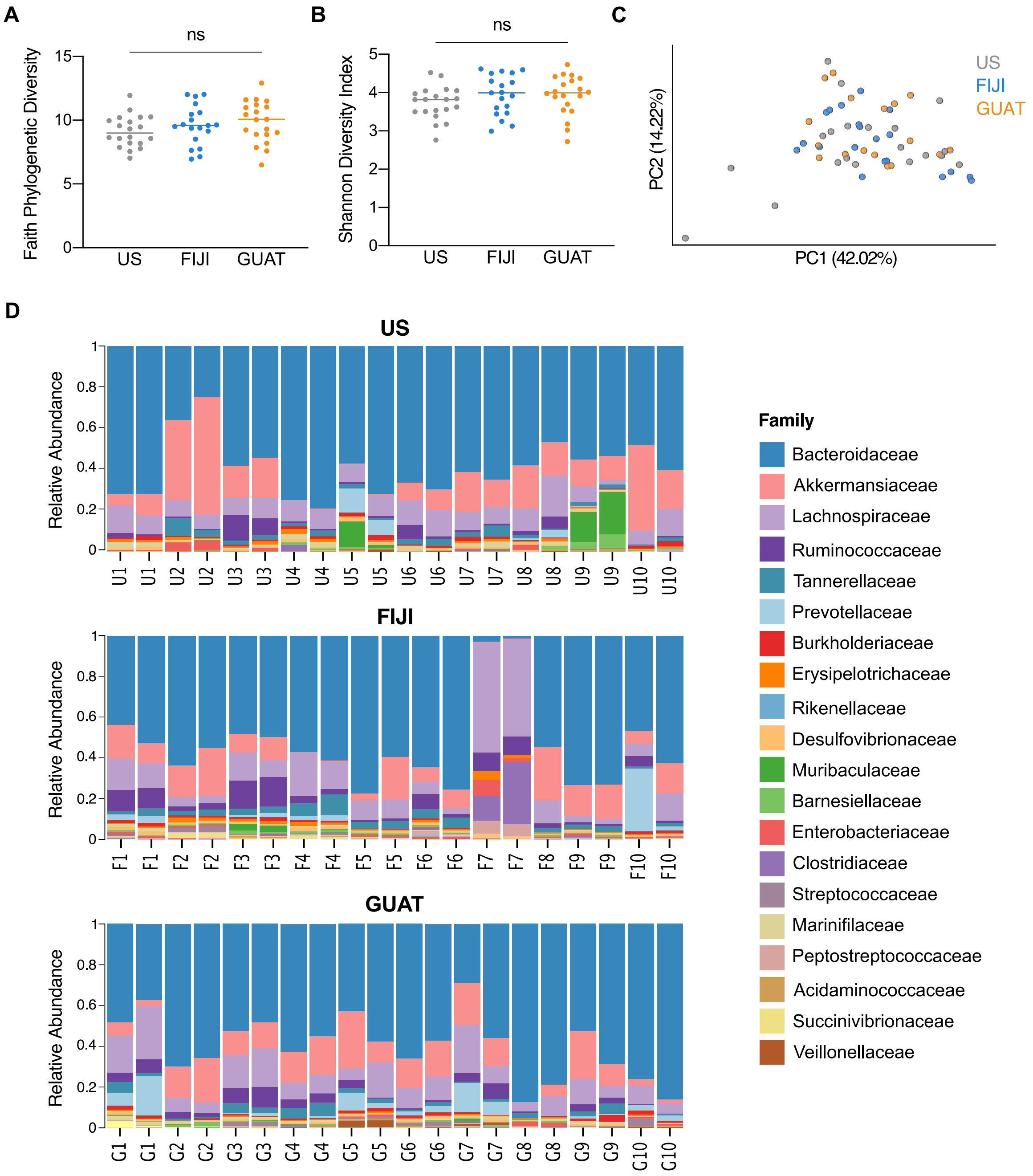


**Supplementary Figure 3. Composition of the US, FIJI, and GUAT mouse microbiomes 14 days post-microbiota transplantation.** Alpha diversity of the mouse gut microbiomes were analyzed through (A) the Faith phylogenetic diversity index and (B) the Shannon diversity index. (C) PCoA of weighted UniFrac distances for the gut microbiomes of the humanized mice 14 days after colonization with donor-associated microbiota. Horizontal bar represents the mean. No statistically significant differences were observed between countries (ns = not significant, one-way ANOVA followed by Tukey's multiple comparison test). (D) Taxa bar plots for all mice grouped at the family level. Each bar represents an individual mouse. Labels on the x-axis denote the original human donor. Related to Figure 1.

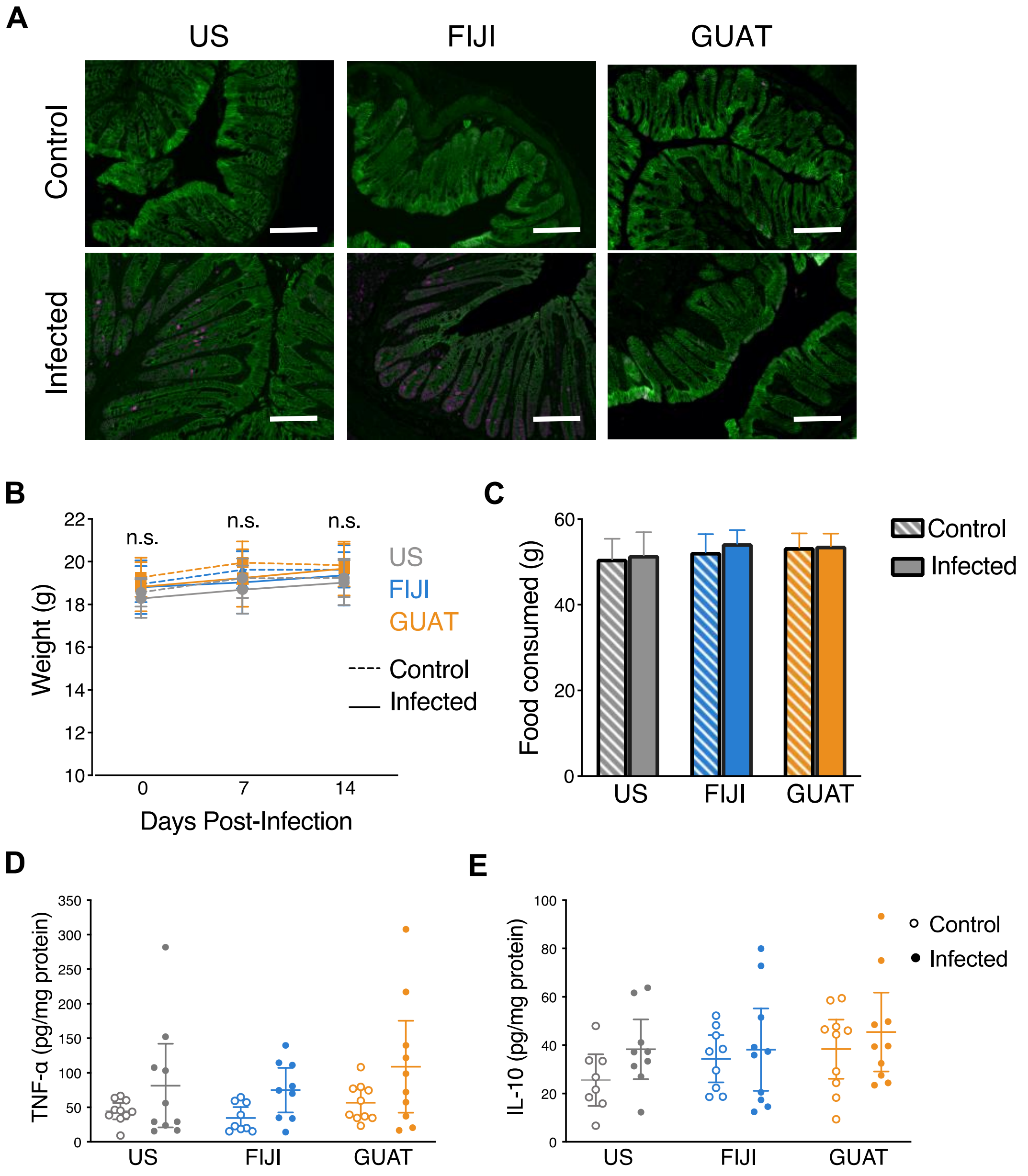


**Supplementary Figure 4. Geographic differences in the microbiome lead to differences in proliferation but not in weight loss or food consumption in response to *C. rodentium* infection.** (A) Representative images of Immunofluorescent staining of ki67, a marker for cell proliferation, in colon sections of control and infected mice 14 days after infection with *C. rodentium*. E-cadherin (cell junctions) is stained green; ki67 is stained magenta. Scale bar represents 100µm. (B) Mouse weight at days 0, 7, and 14 post-infection for all experimental groups. (C) Food consumption between inoculation with *C. rodentium* and 14 days post-infection. (D-E) Quantification of the production of the inflammatory cytokines (D) TNF-α, and (E) IL-10 in *ex vivo* colon culture. Data are presented as mean values ± SD, n.s. = not significant for comparisons shown (one-way ANOVA followed by Tukey's multiple comparison test). Related to Figure 1.

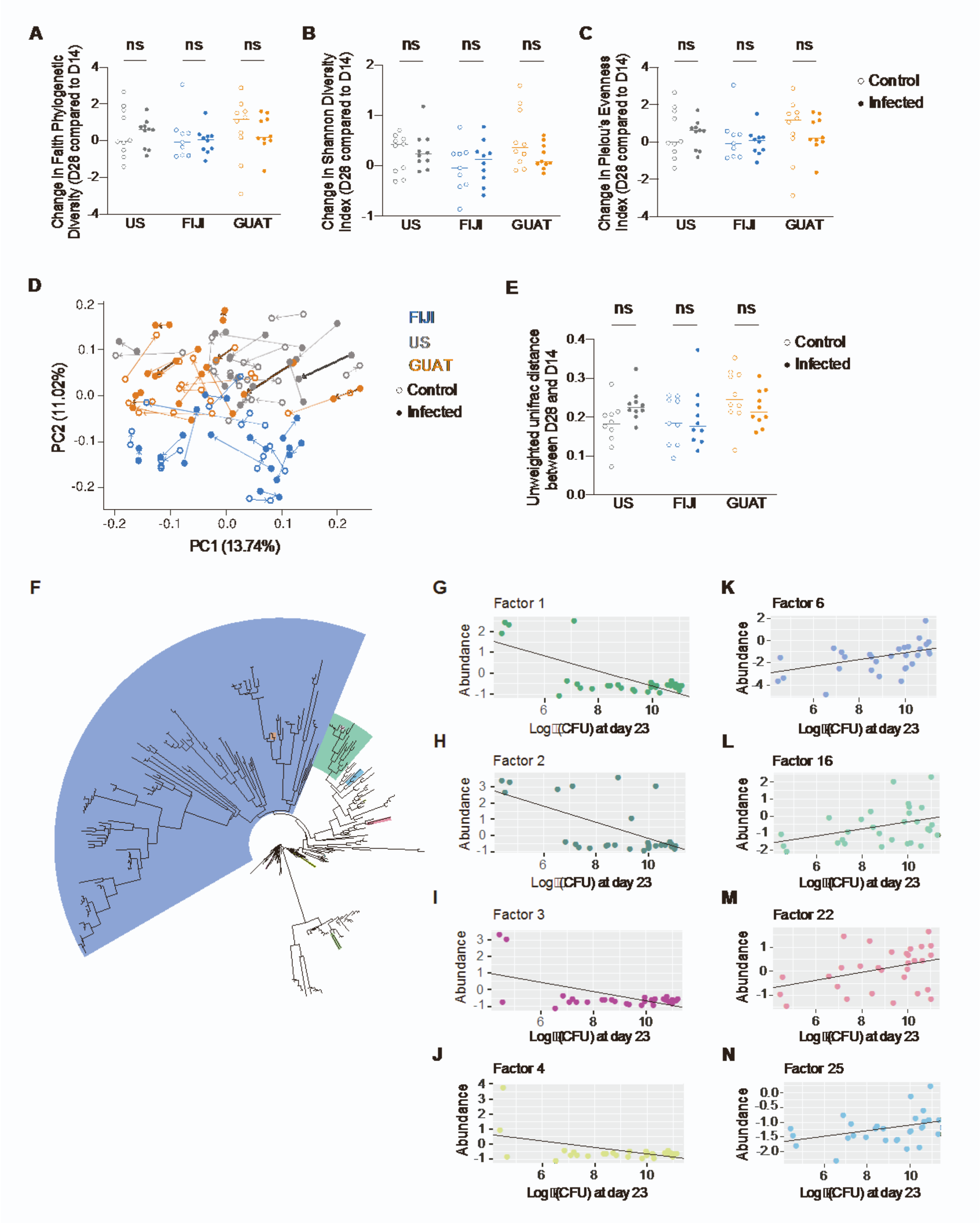


**Supplementary Figure 5. Characterization of the changes in the mouse microbiomes after *C. rodentium* infection.** Quantification of the changes in (A) the Faith phylogenetic diversity index, (B) the Shannon diversity index and (C) Pielou's evenness index before and after infection. (D) PCoA of unweighted UniFrac distances for the gut microbiomes of the humanized mice 14 (pre-infection) and 28 (end of the experiment) days after colonization with

donor-associated microbiota. Arrows originate at day 14 and end at day 28 for the corresponding sample. (E) Quantification of the unweighted Unifrac distance between days 28 and day 14. Horizontal lines represent the mean, ns = not significant for comparisons shown (one-way ANOVA followed by Tukey's multiple comparison test). We also attempted to identify the phylogenetic factors driving *C. rodentium* colonization and shedding using PhyloFactor. (F) Phylogenetic tree of all the ASVs identified across all microbiomes, different colors represent the biggest clades identified as important factors in the analysis. Groups that consist of a single tip are not depicted. (G-N) Linear models depicting the association between specific factors and bacterial shedding at day 23. The line represents predicted values and the dots observed abundances. Related to Figure 1.

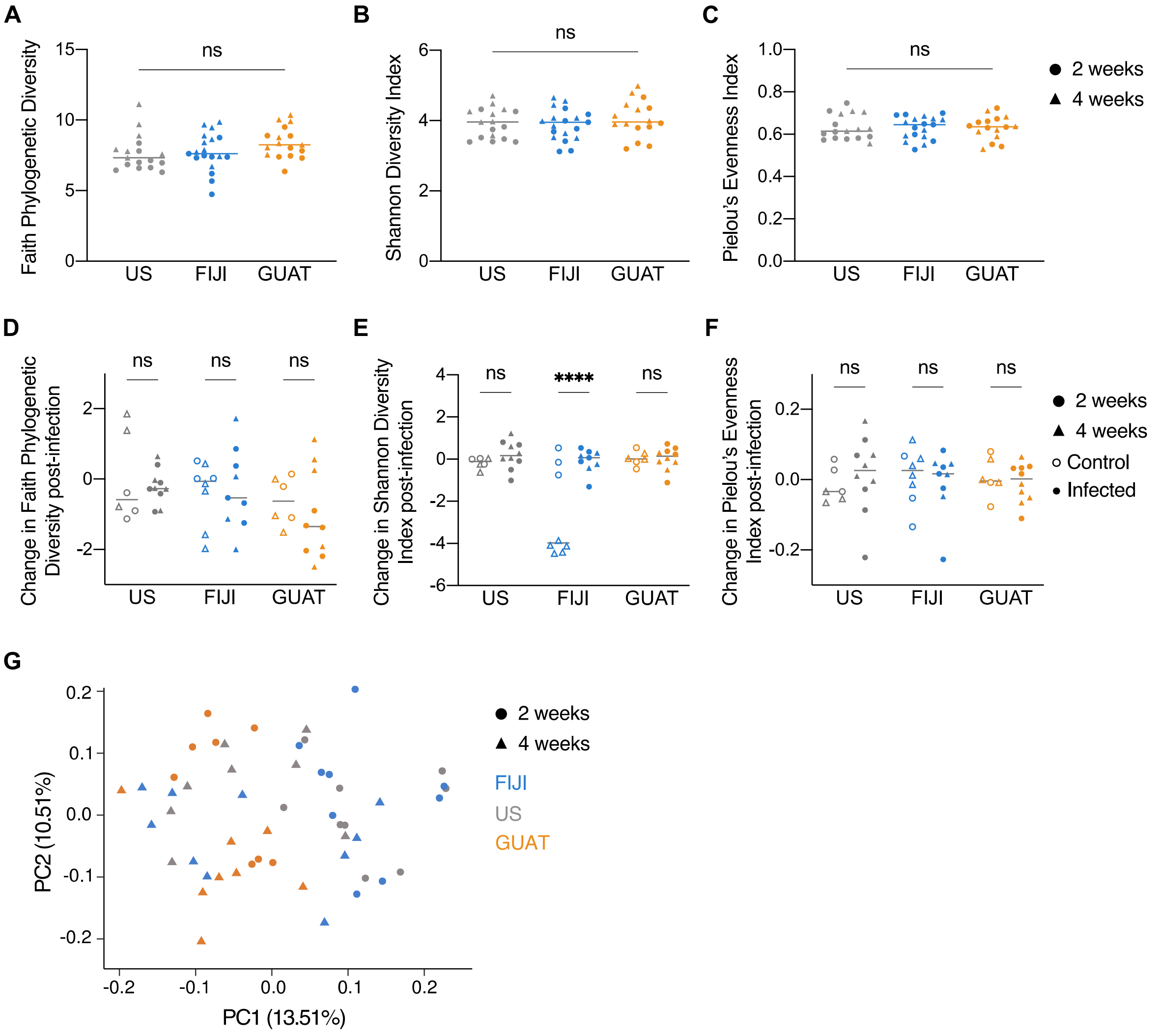


**Supplementary Figure 6. Analysis of the US, FIJI, and GUAT mouse microbiomes before and after infection with *L. monocytogenes*.** Alpha diversity of the mouse gut microbiomes 14 days after microbiota transplantation was analyzed through (A) the Faith phylogenetic diversity index, (B) the Shannon diversity index and (C) Pielou's evenness index. Changes in alpha diversity metrics were also quantified by comparing the microbiomes right before the infection (at days 14 for the 2-week experiment or day 28 for the 4-week experiment) and three days later. Differences in (D) the Faith phylogenetic diversity index, (E) the Shannon diversity index and (F) Pielou's evenness index were calculated. Horizontal lines represent the mean, ****$p<0.0001$, ns = not significant for comparisons shown (one-way ANOVA followed by Tukey's multiple comparison test). (G) PCoA of unweighted UniFrac distances for the gut microbiomes of the humanized mice immediately prior to infection. Related to Figure 3.

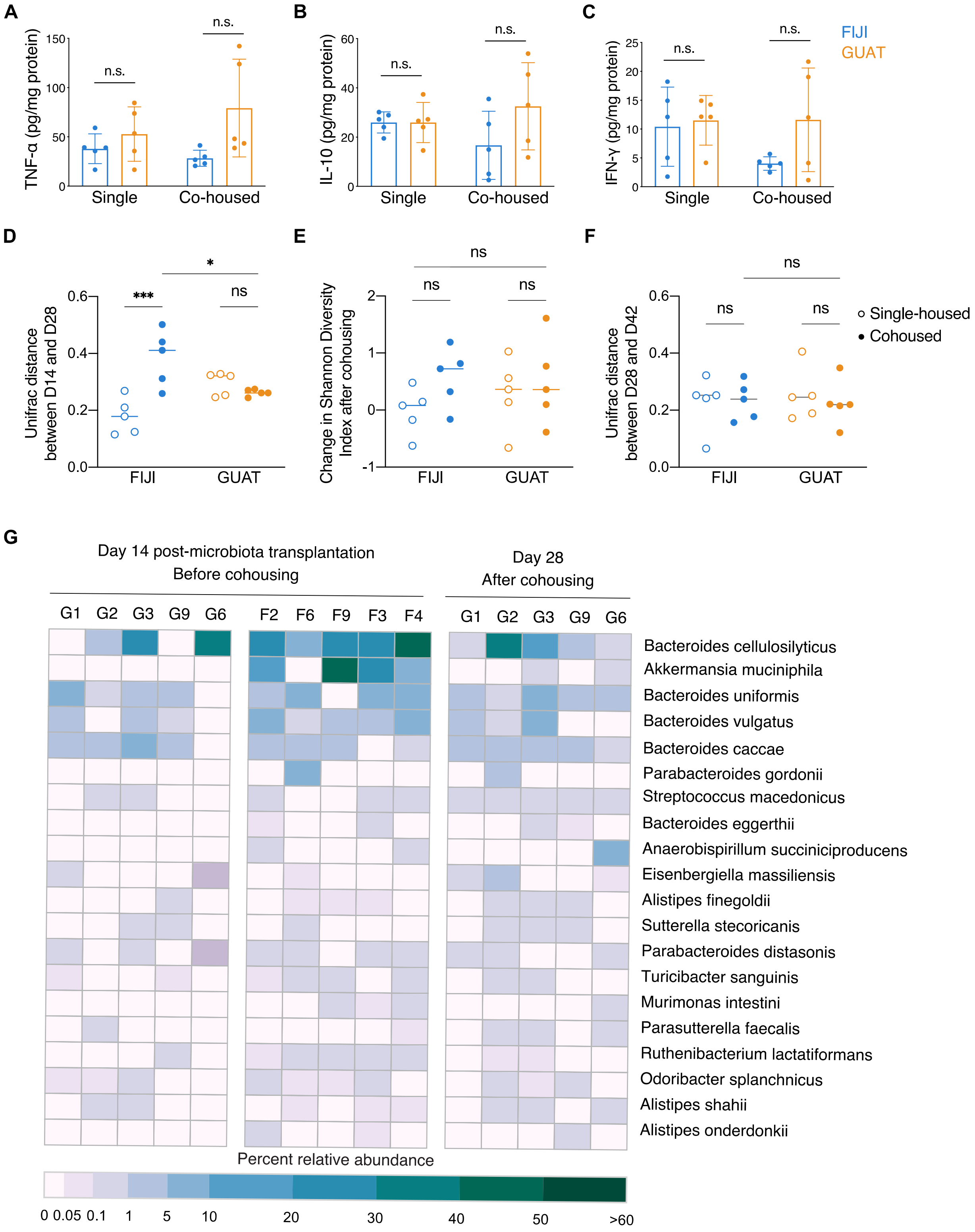


**Supplementary Figure 7. Quantification of changes in inflammatory cytokine secretion and microbiota for all mice after cohousing and infection with *C. rodentium*.** (A-C) Quantification of the production of the inflammatory cytokines (A) TNF-α, (B) IL-10, and (C) IFN-γ in *ex vivo* colon culture. Quantification of (D) the Unifrac distance of

each mouse's microbiome between days 14 and 28 – cohousing, (E) changes in the Shannon diversity index, between days 14 and 28 as a result of cohousing, and (F) Unifrac distance of each mouse's microbiome between (J) days 28 and 42 – infection. Horizontal lines represent the mean with standard deviation were depicted; ns = not significant, *$p<0.05$, ***$p<0.001$ for comparisons shown (one-way ANOVA followed by Tukey's multiple comparison test). Related to Figures 4 and 5.

**Supplementary Table 1. Differentially abundant families between donor and population m**

No differentially abundant families were identified in the Guatemalan cohort. Related to Figure 1

| Percentile abundances of differentially abundant families in the US cohort | | | | | | | | | | |
|---|---|---|---|---|---|---|---|---|---|---|
| | Donors | | | | | Population | | | | |
| **Family** | **0** | **25** | **50** | **75** | **100** | **0** | **25** | **50** | **75** | **100** |
| Succinivibrionaceae | 1 | 1 | 2 | 5.5 | 15 | 1 | 1 | 1 | 1 | 1 |
| Streptococcaceae | 1 | 1 | 8 | 11.25 | 32 | 1 | 18.5 | 27 | 183 | 652 |
| Erysipelotrichaceae | 10 | 16 | 91.5 | 181 | 518 | 41 | 194 | 412 | 950 | 2995 |
| Desulfovibrionaceae | 1 | 6.25 | 12 | 30.25 | 55 | 1 | 26.75 | 87.5 | 125 | 469 |
| Lachnospiraceae | 495 | 1204 | 2635 | 3554 | 14968 | 2735 | 5099 | 8014 | 11044 | 18020 |
| Anaeroplasmataceae | 1 | 1 | 1 | 1 | 4 | 1 | 1 | 1 | 1 | 1 |
| Elusimicrobiaceae | 1 | 1 | 1 | 1 | 3 | 1 | 1 | 1 | 1 | 1 |
| Tannerellaceae | 1 | 19.25 | 73 | 174.3 | 414 | 1 | 237.8 | 385.5 | 676 | 1383 |

| Percentile abundances of differentially abundant families in the Fiji cohort | | | | | | | | | | |
|---|---|---|---|---|---|---|---|---|---|---|
| | Donors | | | | | Population | | | | |
| **Family** | **0** | **25** | **50** | **75** | **100** | **0** | **25** | **50** | **75** | **100** |
| Molicutes | 1 | 8.5 | 16.5 | 51.25 | 616 | 1 | 1 | 1 | 5.75 | 158 |

**Supplementary Table 3. Area under the *C. rodentium* shedding curve ranked in descending order.** Related to Figure 4.

| Mouse ID | Country | AUC |
|---|---|---|
| 230 | Fiji | 3.686E+11 |
| 218 | Fiji | 2.7765E+11 |
| 220 | Fiji | 2.4156E+11 |
| 225 | Fiji | 2.2443E+11 |
| 327 | Fiji | 1.7446E+11 |
| 311 | Fiji | 1.2829E+11 |
| 222 | Fiji | 1.2425E+11 |
| 315 | Fiji | 7.1891E+10 |
| 226 | US | 6.3441E+10 |
| 303 | Fiji | 4.9811E+10 |
| 206 | US | 3.4548E+10 |
| 322 | US | 2.5877E+10 |
| 329 | US | 2.4714E+10 |
| 305 | US | 1.6616E+10 |
| 325 | Guatemala | 1.0298E+10 |
| 319 | Guatemala | 7050120000 |
| 221 | US | 6999058300 |
| 310 | Guatemala | 4575879300 |
| 318 | US | 3954273350 |
| 213 | Guatemala | 3948202000 |
| 205 | US | 2218280000 |
| 211 | US | 1843546800 |
| 309 | Fiji | 1505363000 |
| 214 | Guatemala | 784928800 |
| 313 | US | 725066950 |
| 229 | Guatemala | 713095000 |
| 204 | Guatemala | 362752660 |
| 324 | Guatemala | 108296500 |
| 330 | Guatemala | 15600600 |
| 212 | Guatemala | 3745600 |
| 313 | US | 713095000 |
