# Supplementary material for: Geographic differences in gut microbiota composition impact susceptibility to enteric infection
Source: Cell Rep. 2021 Jul 27;36(4):109457. doi: 10.1016/j.celrep.2021.109457 (PMC8333197; doi:10.1016/j.celrep.2021.109457)
Supplement: Document S2. Article plus supplemental information [file mmc5.pdf]

Cell Reports

Article

Geographic differences in gut microbiota composition impact susceptibility to enteric infection

Graphical abstract

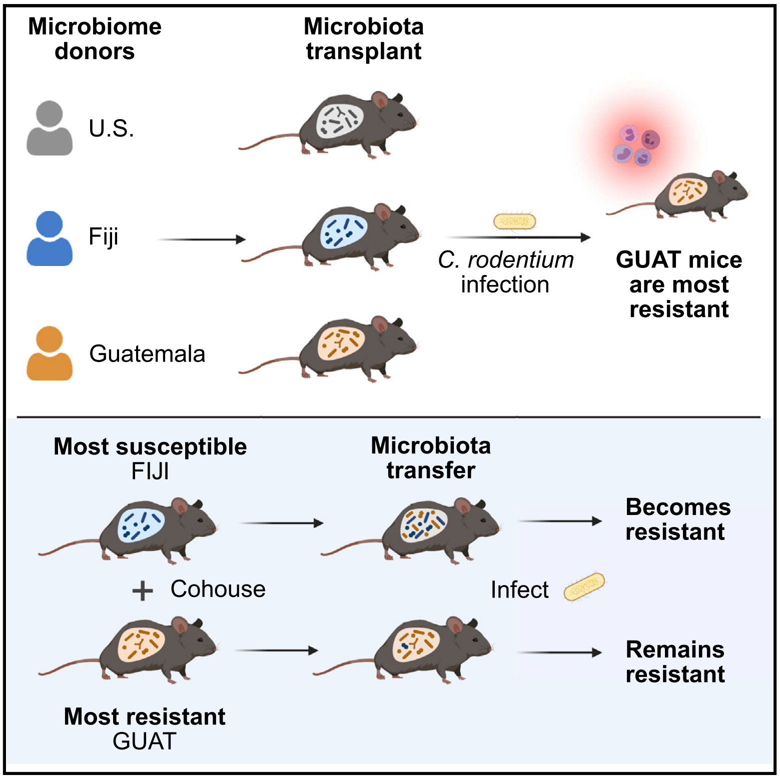

Highlights

- Germ-free mice were humanized with microbiota from three global populations
- Mice exhibit geography-specific susceptibilities to *Citrobacter rodentium* infection
- Resistance to *C. rodentium* infection may be driven by high basal inflammation levels
- Co-housing mice from different geographies improves resistance to infection

Authors

Ana Maria Porras, Qiaojuan Shi, Hao Zhou, Rowan Callahan, Gabriella Montenegro-Bethancourt, Noel Solomons, Ilana Lauren Brito

Correspondence

ibrito@cornell.edu

In brief

Significant differences in gut microbiome composition distinguish human populations around the globe. Using germ-free mice, Porras et al. show that microbial differences alone can impact immune responses and susceptibility to enteric infection. They show that, by co-housing mice humanized with microbiomes from different geographies, they can transfer resistance to infection.

Porras et al., 2021, Cell Reports *36*, 109457
July 27, 2021 © 2021 The Author(s).
https://doi.org/10.1016/j.celrep.2021.109457

CellPress

Cell Reports

CellPress
OPEN ACCESS

Article

Geographic differences in gut microbiota composition impact susceptibility to enteric infection

Ana Maria Porras,[1] Qiaojuan Shi,[1] Hao Zhou,[2] Rowan Callahan,[3] Gabriella Montenegro-Bethancourt,[4] Noel Solomons,[4] and Ilana Lauren Brito[1,5,*]
[1]Meinig School of Biomedical Engineering, Cornell University, Ithaca, NY, USA
[2]Department of Microbiology, Cornell University, Ithaca, NY, USA
[3]Cancer Early Detection Advanced Research Center, Oregon Health & Science University, Portland, OR, USA
[4]Center for Studies of Sensory Impairment, Aging and Metabolism (CeSSIAM), Guatemala City, Guatemala
[5]Lead contact
*Correspondence: ibrito@cornell.edu
https://doi.org/10.1016/j.celrep.2021.109457

SUMMARY

Large-scale studies of human gut microbiomes have revealed broad differences in composition across geographically distinct populations. Yet, studies examining impacts of microbiome composition on various health outcomes typically focus on single populations, posing the question of whether compositional differences between populations translate into differences in susceptibility. Using germ-free mice humanized with microbiome samples from 30 donors representing three countries, we observe robust differences in susceptibility to *Citrobacter rodentium*, a model for enteropathogenic *Escherichia coli* infections, according to geographic origin. We do not see similar responses to *Listeria monocytogenes* infections. We further find that cohousing the most susceptible and most resistant mice confers protection from *C. rodentium* infection. This work underscores the importance of increasing global participation in microbiome studies related to health outcomes. Diverse cohorts are needed to identify both population-specific responses to specific microbiome interventions and to achieve broader-reaching biological conclusions that generalize across populations.

INTRODUCTION

Mechanistic studies have provided causal linkages between the human gut microbiome and diseases ranging from inflammatory bowel disease (Britton et al., 2019; Ni et al., 2017) to atherosclerosis (Koeth et al., 2013), obesity (Ridaura et al., 2013), diabetes (Uusitalo et al., 2016; Zhang et al., 2020), and mental disorders (Zheng et al., 2016, 2019). However, these studies have primarily involved human subjects from high-income and upper-middle-income nations across North America, Europe, and Asia (Almeida et al., 2019; Brewster et al., 2019; Gupta et al., 2017; Magne et al., 2016; Nayfach et al., 2019; Olesen et al., 2020; Pasolli et al., 2019; Porras and Brito, 2019). In spite of the growing appreciation for the large-scale differences in the composition of microbiomes throughout the world (Almeida et al., 2019; Brito et al., 2016; De Filippo et al., 2010; He et al., 2018; Lin et al., 2013; Martínez et al., 2015; Nayfach et al., 2019; Pasolli et al., 2019; Yatsunenko et al., 2012; Zhong et al., 2019), studies causally linking the microbiome to health and disease outcomes have not broadly considered the global diversity of human gut microbiomes. This unexplored diversity in the composition of the gut microbiome can have important consequences for immunological function in the host (Sonnenburg and Sonnenburg, 2019).

The microbiome factors significantly into the regulation of immune homeostasis, and alterations of the gut microbiome can lead to the development of autoimmune and metabolic disorders, altered innate and adaptive immune responses to infection, and inflammation (Libertucci and Young, 2019; Wu and Wu, 2012). Furthermore, there is supporting evidence that the organisms that differ in their global distribution may contribute to promoting or reducing inflammation and maintaining homeostasis. For example, mucus-degrading species abundant in high-income countries like those of the *Bacteroides* and *Akkermansia* genera have been linked to anti-inflammatory properties and improved metabolic function (Brown et al., 2019; Plovier et al., 2017; Png et al., 2010). Species of the *Prevotella* genus, which dominate many microbiomes in low- and middle-income countries, have been linked to improved glucose metabolism in Swedish adults (Kovatcheva-Datchary et al., 2015). Yet, both the *Prevotella* and *Bacteroides* genera have been associated in other clinical and mouse studies with rheumatoid arthritis (Maeda et al., 2016; Scher et al., 2013), hypertension (Scher et al., 2013), obesity (Hu et al., 2015), and inflammatory bowel disease (Hickey et al., 2015; Saitoh et al., 2002) in high-income settings. Given these conflicting results, it is very difficult to translate the roles of specific members of each population's

This is an open access article under the CC BY license (http://creativecommons.org/licenses/by/4.0/).

microbiomes and rather, consideration of geographic differences in gut microbiota composition on host health may be more relevant in the context of global health.

In particular, diarrheal and enteric diseases remain a leading cause of mortality with low- and middle-income countries bearing most of the burden (GBD 2016 Diarrheal Disease Collaborators, 2018). Although many factors (host genetics, exposure to pathogens, sanitation infrastructure, delivery of clinical services, etc.) may contribute to population-level susceptibility to enteric infections (Mandeville et al., 2009), reports from populations around the world have demonstrated the crucial role of the gut microbiome in providing protection against exogenous enteropathogens. Fecal microbiota transplants in Italian, US, Canadian, and Dutch populations have proved effective for the treatment of *Clostridioides difficile* infections (Ianiro et al., 2018; Jiang et al., 2017; Lee et al., 2016; van Nood et al., 2013). Moreover, an individual's gut microbiome composition can predict colonization by gut parasites like *Entamoeba* and soil-transmitted helminths in rural African populations (Morton et al., 2015; Rubel et al., 2020), and by *Vibrio cholera* in a pediatric Bangladeshi cohort (Midani et al., 2018). These seminal reports highlight the importance of exploring the causal relationship between the gut microbiome and enteric infections while considering global differences in microbiomes.

Studies in mice provide further evidence that gut microbes mediate colonization resistance to prevent infections (Libertucci and Young, 2019; McKenney and Pamer, 2015). For example, a metabolite produced by *Bacteroides* species inhibits colonization by *Salmonella enterica* (Jacobson et al., 2018), and, in both immune-deficient Raggc and chemotherapy-treated mice, gut microbes provide a defense against *Listeria monocytogenes* infection (Becattini et al., 2017). Similarly, changes in the microbiome can lead to increased pathogen colonization in mouse models of *Escherichia coli* infections (Bailey et al., 2010; Mullineaux-Sanders et al., 2017). Gut microbes regulate colonization by enteropathogens not only through direct interspecies competition but also through modulation of the host's immune system (Caballero and Pamer, 2015). Commensal gut microbes have the capacity to induce interleukin (IL)-22- and Th17-dependent immune responses to enteric infections (Ivanov et al., 2008; Willing et al., 2011). Nonetheless, studies that explore the causal relationship between the gut microbiome and bacterial enteropathogens have not yet incorporated cohorts that test the impact of global variation in gut microbiome composition. We hypothesized that differences in the human microbiome, largely defined by geography, may underlie differences in susceptibility to enteric infection.

To test this hypothesis, we sought to transplant stool samples from three geographically distinct populations into germ-free (GF) mice. We chose to compare individuals from an urban population in Guatemala, a suburban population in the United States, and an agrarian community in the Fiji Islands. Humanized GF mice have been used extensively to study the causal relationship between gut microbiota and disease state (Baxter et al., 2014; Berer et al., 2017; Chiu et al., 2017; De Palma et al., 2017; Ridaura et al., 2013; Turnbaugh et al., 2006). Often, the ability of these models to capture the inter-individual variability of the human microbiome is limited by small donor numbers (one to five) or pseudo-replication of animal specimens (Walter et al., 2020). This pseudo-replication arises when stool from a single donor or pooled donors is transplanted into multiple mice that are later analyzed as individual biological units, thus inflating the biologically relevant sample size. To address these issues, we included 30 individual donors (10 per country), whose microbiota were individually transplanted into separate mice such that the individual biological replicates were conserved. Following colonization of the mouse guts, the animals were challenged with two enteric pathogens: (1) *Citrobacter rodentium*, to model enteropathogenic and enterohermmorrhagic *E. coli* infections (Crepin et al., 2016) and (2) *L. monocytogenes.* This approach allowed us to evaluate whether microbiome differences impact susceptibility to enteric infection in mice with microbiomes representative of those found in populations in the United States, Fiji, and Guatemala.

RESULTS

Humanized gnotobiotic mice reflect their human donors and respective populations

We first chose human donors in the United States, Fiji, and Guatemala whose microbiomes were representative of their respective populations. We identified suitable donors within a cohort of Guatemalan donors from the city of Quetzaltenango recruited for this study and a subset of 36 donors from the Fiji Community Microbiome Project (Brito et al., 2016) so that they were gender and age (within 5 years) matched. For the subjects from the United States, we recruited donors with similar demographics and compared them to publicly available gut microbiome data from the same country (Yatsunenko et al., 2012). We selected a total of 10 donors (five male and five female) per country between the ages of 23 and 43 with mean ages of 32.1, 32.8, and 31.2 years old for the American, Fijian, and Guatemalan donors, respectively (Table 1). As expected based on previously published studies (Brito et al., 2016; Yatsunenko et al., 2012), after rarefying the samples and performing community-level analyses, we observed strong separation between the cohorts (Figure 1A) and significant differences in phylogenetic diversity between countries but not between donors and their respective populations (Figures S1A–S1C). Additionally, the taxonomic composition of the selected donor microbiomes was similar to that observed in other subjects within their respective populations (Figure S1D). More specifically, analysis of composition of microbes (Mandal et al., 2015) at the family level revealed eight differentially abundant families between US donors and the population, most of which are of low relative abundance (Table S1). Only one differentially abundant family between Fijian donors and population were identified, and no differences were observed between the Guatemalan groups. In this way, we confirmed that the selected donor microbiomes were representative of their respective population microbiomes.

We next evaluated whether the microbiomes of the humanized gnotobiotic mice reflected those of their human donors. Mice whose guts were colonized with microbiota from the same donor were housed in pairs prior to inoculation with pathogenic microorganisms. A paired analysis (Fouladi et al., 2020) revealed that 53.6% ± 12.7%, 30.6% ± 13.6%, and 51.8% ± 12.6% of all amplicon sequence variants (ASVs) present in the US, FIJI, and

Table 1. Metadata of the human donors selected for this study

United States			Fiji			Guatemala		
DonorID	Gender	Age	DonorID	Gender	Age	DonorID	Gender	Age
U1	F	28	F1	F	26	G1	F	27
U2	F	35	F2	F	34	G2	F	37
U3	M	23	F3	M	25	G3	M	23
U4	F	24	F4	F	26	G4	F	24
U5	M	29	F5	M	27	G5	M	27
U6	F	33	F6	F	35	G6	F	32
U7	M	34	F7	M	35	G7	M	31
U8	M	34	F8	M	40	G8	M	32
U9	M	43	F9	M	40	G9	M	39
U10	F	38	F10	F	40	G10	F	40

Ten donors per country were selected after matching for age and gender.

GUAT mice, respectively, originated from their corresponding human donor (Figure S2A). This limited colonization by human donors is comparable to that reported by others in similar studies using gnotobiotic mice (Fouladi et al., 2020). After considering all ASVs in the dataset, we were able to identify that, on average, the remaining ASVs likely originated from other donors in the same country (24.1% ± 12.7% for all mice) and to a lesser extent, from donors in other countries (12.2% ± 8.6%). A small proportion of all mouse microbiomes was made up of ASVs identified only in the mice (Figures S2B–S2D; Table S2). It is important to note that even though the mice were housed in sterile filter-top cages and handled with sterile technique, rather than in gnotobiotic isolators, their microbiomes remained relatively stable over the course of the 4-week experiment (Figure S5).

To further understand the composition of the humanized mouse microbiomes, it was necessary to move beyond a presence/absence analysis and quantify the relative abundances of these ASVs. We identified that most of the US (85.3% ± 7.7%) and GUAT (70.7% ± 20.2%) microbiomes are composed of ASVs that were successfully transferred from the corresponding donor (Figures S2B–S2D). While colonization of the FIJI guts by microbiota associated with the corresponding donor was less successful (37.6% ± 18.1%), most of the ASVs in these microbiomes originated from either the original donor or another Fijian donor (total of 77.82% ± 13.4%). Not all bacterial phyla exhibit the same colonization efficiency. Taxa belonging to the *Proteobacteria* and *Bacteroidetes* phyla were better able to colonize mouse guts compared to those in the *Firmicutes* phylum (Figure S2E). No statistically significant differences in phylogenetic diversity were found across these mouse cohorts (Figures S3A and S3B). An analysis of the beta diversity across experimental groups revealed separation between them similar to that observed in the human donors (Figure 1B).

Geographic differences in the microbiome lead to differences in susceptibility to *C. rodentium* infection in humanized mice

To determine whether geographic differences in the human microbiome lead to differences in susceptibility to enteric infection, we challenged the mice with *C. rodentium* (1×10^9 CFU) or PBS 14 days after colonization of their guts with human-associated microbiota (Figure 1C). Monitoring of *C. rodentium* shedding in the stool after inoculation revealed an infection peak at day 5 for the GUAT mice compared to day 9 for both the US and FIJI mice (Figure 1D). Moreover, the GUAT mice exhibited significantly lower bacterial shedding than the FIJI and US mice starting at days 7 and 9 post-infection, respectively, with the FIJI mice presenting the highest levels of shedding throughout the course of the experiment. These differences in *C. rodentium* shedding between countries are also reflected in the AUC values calculated over 14 days post-infection (Figure 1E). Differences in susceptibility according to the geography of the donors were confirmed by histological analysis of explanted colons 14 days post-infection, where evidence of colonic crypt hyperplasia was observed in the US and FIJI mice but not in the GUAT mice (Figures 1F and 1G). Similar differences in cell proliferation in the colon post-infection were also observed through staining of the ki67 marker (Figure S4A). No significant differences in mouse weight and food consumption between countries were identified at any point of the experiment (Figures S4B and S4C).

Basal inflammation levels in the GUAT mice may drive resistance to *C. rodentium* infection

To assess the role of inflammation in these differences, we quantified lipocalin-2 (LCN-2) and calprotectin levels in the mouse stool at the end of the experiment. Elevated levels of LCN-2 were found in the stool when comparing infected to control mice in all three cohorts, as expected after a pathogenic infection (Figure 2A). Calprotectin levels in contrast were only significantly elevated in the FIJI infected mice compared to the controls (Figure 2B). In both cases, GUAT control mice exhibited higher LCN-2 and calprotectin levels compared to their US and FIJI counterparts. We also evaluated the secretion of several inflammatory cytokines known to be involved in the defense against *C. rodentium* through *ex vivo* culture of the colon at the end of the experiment (Atarashi et al., 2015; Basu et al., 2012; Chen et al., 2015; Ivanov et al., 2009). Due to the financial and logistical limitations of germ-free mouse experiments, we were not able to assess the production of these cytokines in the early stages or at the peak of the infection, when they would be expected to be at their highest levels (Silberger

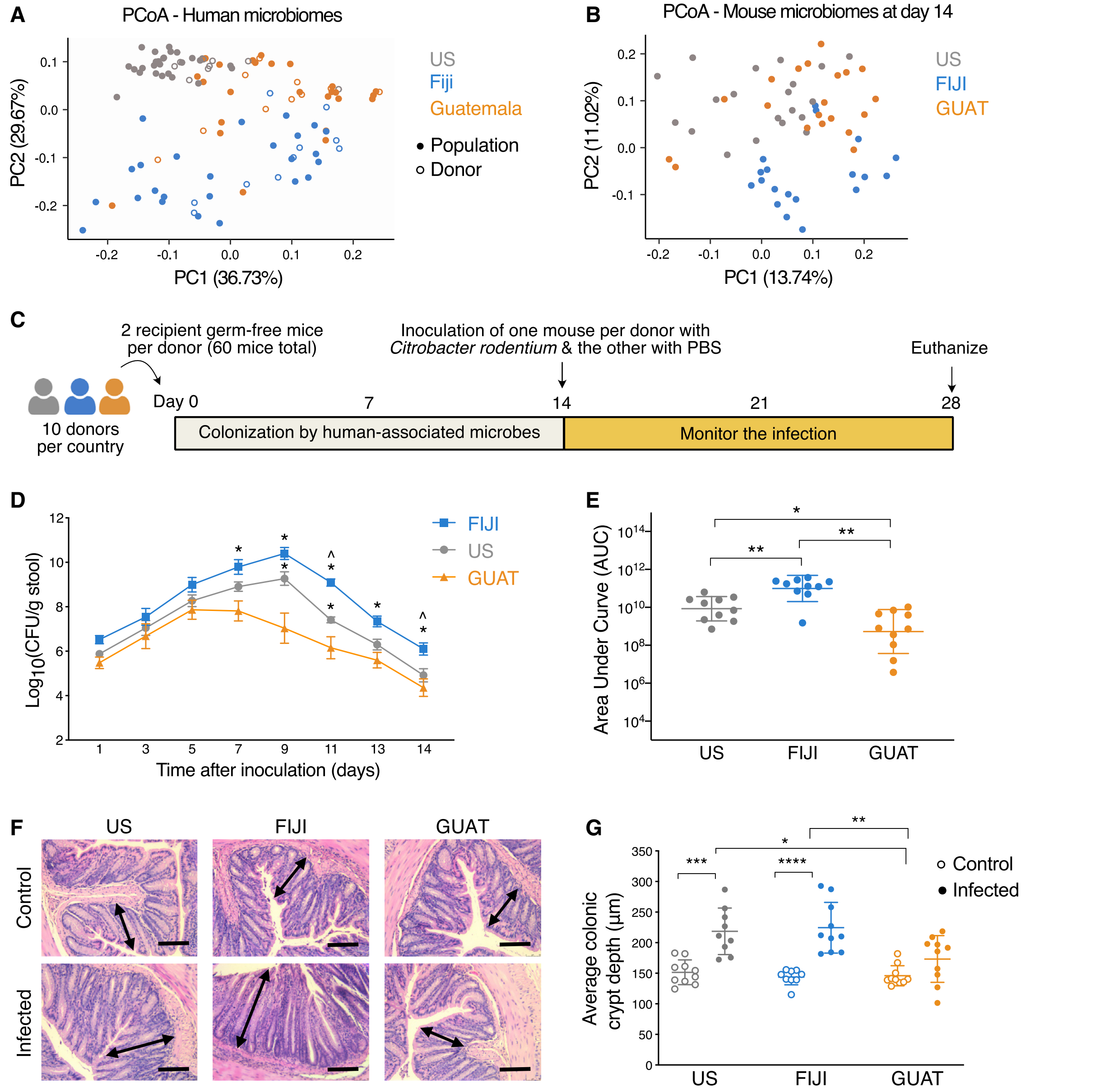

Figure 1. Geographic differences in the microbiome lead to differences in susceptibility to *C. rodentium* infection in humanized mice

(A) PCoA of weighted UniFrac distances for the gut microbiomes of subjects from their respective populations including the human donors.

(B) PCoA of unweighted UniFrac distances for the gut microbiomes of the humanized mice 14 days after colonization with donor-associated microbiota.

(C) Experimental design. We obtained stool samples from 10 donors per country. Two germ-free mice received microbiota transplants from a single human donor within 24 h of arrival at our facility. For each pair of mice associated with a single donor, one mouse was inoculated with *C. rodentium* and the other with PBS. The infection was monitored for the following 14 days.

(D) Quantification of *C. rodentium* shedding in stool. Data are presented as mean ± SEM and n = 10 mice per country from two independent experiments. *$p < 0.05$ compared to GUAT mice at the same time point and ^$p < 0.05$ compared to US mice at the same time point (repeated-measures two-way ANOVA followed by Tukey's multiple comparison test).

(E) Quantification of the area under the *C. rodentium* shedding curve.

(F) Representative images of H&E-stained colon sections of control and infected mice 14 days after infection. The arrows depict average crypt length with hyperplasia observed in the US and FIJI mice. Scale bar represents 100 µm.

(G) Quantification of average colonic crypt depth in control and infected mice 14 days after infection.

For (E)–(G), data are presented as mean values ± SD, with *$p < 0.05$, **$p < 0.01$, ***$p < 0.001$, ****$p < 0.0001$ for comparisons shown (two-way ANOVA followed by Tukey's multiple comparison test). See also Figures S1–S5.

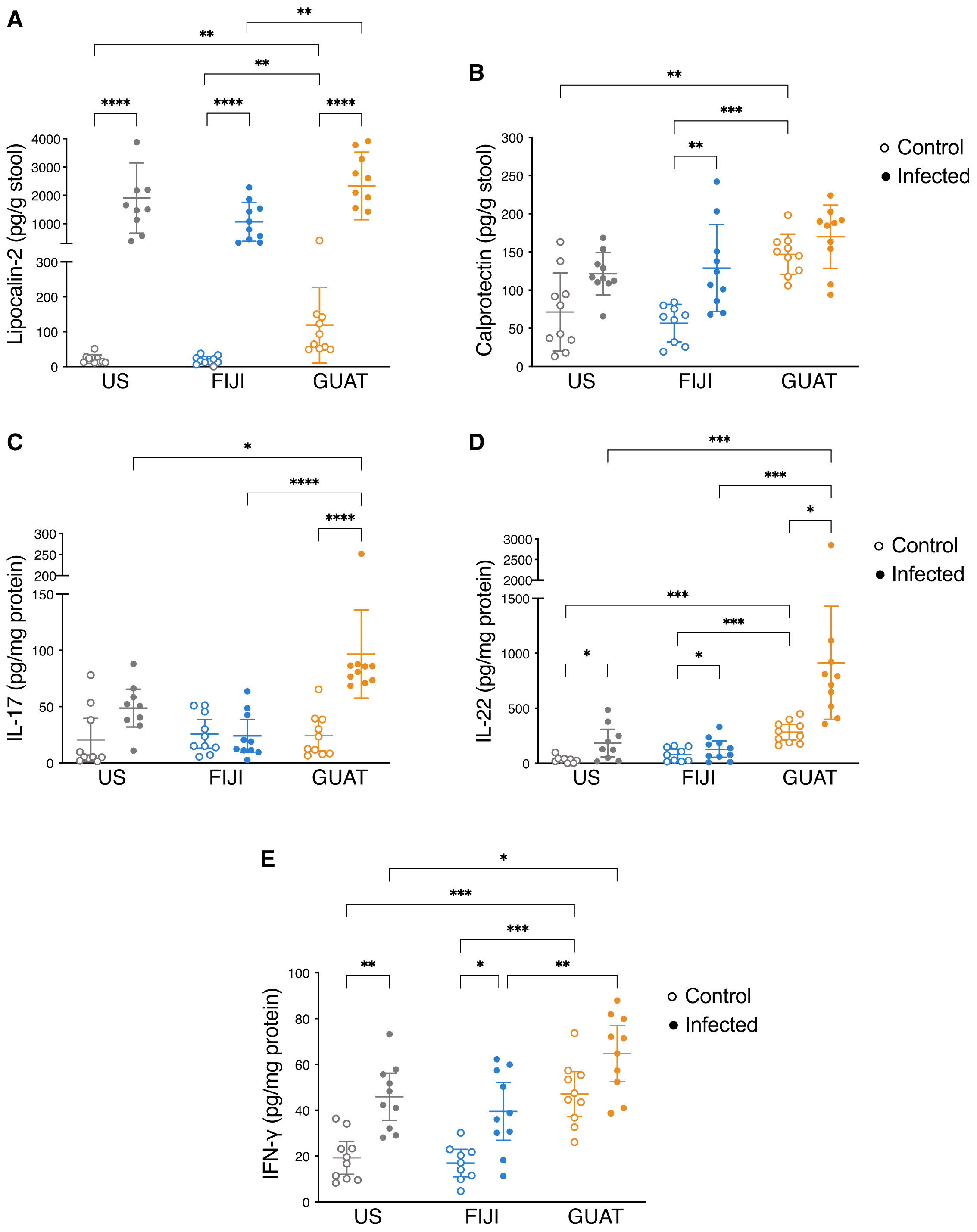

Figure 2. Basal inflammation levels in the GUAT mice may drive resistance to *C. rodentium* infection

(A and B) Assessment of (A) lipocalin-2 and (B) calprotectin levels in the stool of control and infected mice 14 days after infection.

(C–E) Quantification of the production of the inflammatory cytokines (C) IL-17, (D) IL-22, and (E) IFN-γ in *ex vivo* colon culture.

Data are presented as mean values ± SD, with $^*p < 0.05$, $^{**}p < 0.01$, $^{***}p < 0.001$, $^{****}p < 0.0001$ for comparisons shown (two-way ANOVA followed by Tukey's multiple comparison test, n = 10 biological replicates). See also Figure S4.

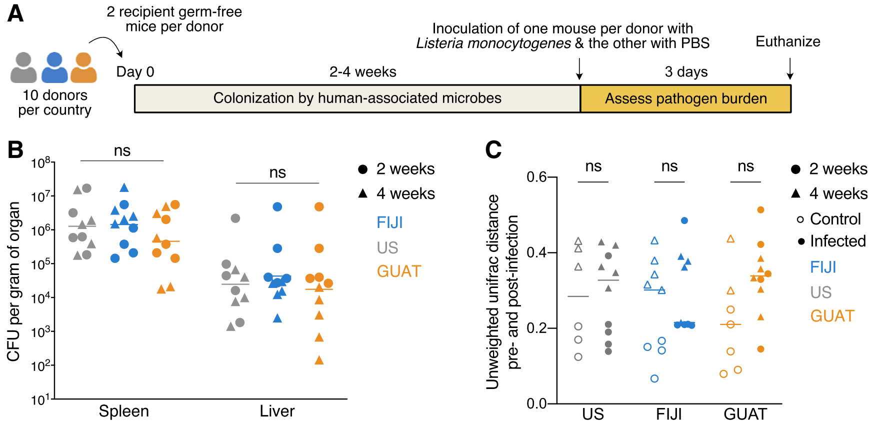

Figure 3. Country-specific microbiome differences in susceptibility to infection do not extend to a Th1 *L. monocytogenes* infection

(A) Experimental design. Using stool samples from the same donors in the *C. rodentium* infection model, two germ-free mice per donor received microbiota transplants within 24 h of arrival at our facility. Two independent experiments were conducted, in which one mouse per pair was infected with *L. monocytogenes*, with the other mouse receiving a PBS mock injection, either 2 or 4 weeks after colonization with human-associated microbiota. Pathogen burden in the liver and spleen were assessed 3 days after inoculation with this pathogen.

(B) Quantification of *L. monocytogenes* burden in the liver and spleen of the humanized mice 3 days after infection. Horizontal line represents the mean.

(C) Unweighted UniFrac distances between samples immediately before and 3 days post-infection. No statistically significant differences were observed between countries (ns, not significant, two-way ANOVA followed by Tukey's multiple comparison test). For all panels, n = 6–10 biological replicates. See also Figure S6.

et al., 2017; Waki et al., 2017). Nonetheless, colonic sections isolated from the infected GUAT mice produced significantly higher amounts of the inflammatory cytokines interleukin-17 (IL-17; Figure 2C), interleukin-22 (IL-22; Figure 2D) and interferon gamma (IFN-γ; Figure 2E) compared to their infected US and FIJI counterparts. No statistically significant differences were observed in the secretion of tumor necrosis factor alpha (TNF-α; Figure S4D) or interleukin-10 (IL-10; Figure S4E). As was the case for LCN-2 and calprotectin, IL-22 and IFN-γ were secreted in higher quantities in the GUAT control mice compared to the other two uninfected mouse cohorts (Figures 2D and 2E). These results indicate a basal level of increased inflammation in the GUAT mice that could explain the resistance against and early clearance of *C. rodentium* in these mice, as well as the continued elevated levels of these molecules in this cohort 14 days after inoculation.

After analyzing the microbiome of all mice, we found no significant changes in microbiome composition after inoculation with either *C. rodentium* or PBS in any of the experimental groups (Figures S5A–S5E). Phylogenetic factorization using PhyloFactor (Washburne et al., 2017) did not identify specific ASVs or clades definitely correlated with severity of infection (Figures S5F–S5N), perhaps due to the inherent variability in the data. Additionally, our microbiome analysis may be incomplete given that it does not contained sequencing of mucosal-associated microbes that are greatly impacted during *C. rodentium* infections (Hoffmann et al., 2009). Overall, the results from this experiment suggest that the GUAT mice are more resistant to *C. rodentium* infection than their US and FIJI counterparts and are able to mount a stronger immune response against this pathogen. Moreover, these differences in susceptibility to infection mediated by the immune system are observed only 2 weeks post-transplantation of human-associated microbiota.

Country-specific microbiome differences in susceptibility to infection do not extend to a Th1 *L. monocytogenes* infection

Many extracellular enteric pathogens like *C. rodentium* and *E. coli* elicit a strong immune response mediated by T-helper 17 (Th17) cells (Atarashi et al., 2015; Collins et al., 2014; Zambrano-Zaragoza et al., 2014). We were interested in determining whether the same geographic effect on susceptibility to infection would be observed when the mice were challenged with a pathogen that primarily elicits a Th1 immune response (Hsieh et al., 1993; Yang et al., 2014). Using microbiota from the same donors as the *C. rodentium* experiments, germ-free mice were humanized in two independent experiments (5 distinct donors per country per experiment, same 10 donors total as the *C. rodentium* experiment; Figure 3A). Half of the mice (one per donor) were then inoculated with *L. monocytogenes* (1×10^4 CFU) and the other half with PBS either 2 or 4 weeks after microbiota transplantation to model an acute systemic infection. No differences in pathogen burden in the liver or spleen were observed across countries in either experiment (Figure 3B). Similarly, the gut microbiomes of these mice did not change significantly post-infection (Figure 3C; Figure S6). These results indicate that the observed differences in susceptibility to infection may be limited to the mucosal Th17 pathway within our experimental setup.

Resistance to *Citrobacter rodentium* infection in susceptible gnotobiotic mice is transferrable after exposure to resistant gnotobiotic mice

We next sought to establish whether resistance to *C. rodentium* infection could be transferred through gut microbiota. First, we identified the 5 most susceptible and 5 most resistant mice to this pathogen in our previous experiment by comparing AUC values for *C. rodentium* shedding in the stool (Table S3). Coincidentally, the most susceptible and most resistant mice were originally gavaged with stool slurries prepared from Fijian and Guatemalan donors, respectively. We then transplanted the cecal contents of each of these 10 mice into 2 germ-free mice per donor. Two weeks after colonization of their guts, one mouse per donor was moved to a different cage to be cohoused with a mouse of the other experimental group for a total of 5 cohoused FIJI and GUAT pairs (Figure 4A). All mice were challenged with *C. rodentium* (1×10^9 CFU) 2 weeks after the beginning of the cohousing window.

As observed in the previous experiment, the single-housed FIJI mice exhibited significantly higher levels of pathogen

CellPress
OPEN ACCESS

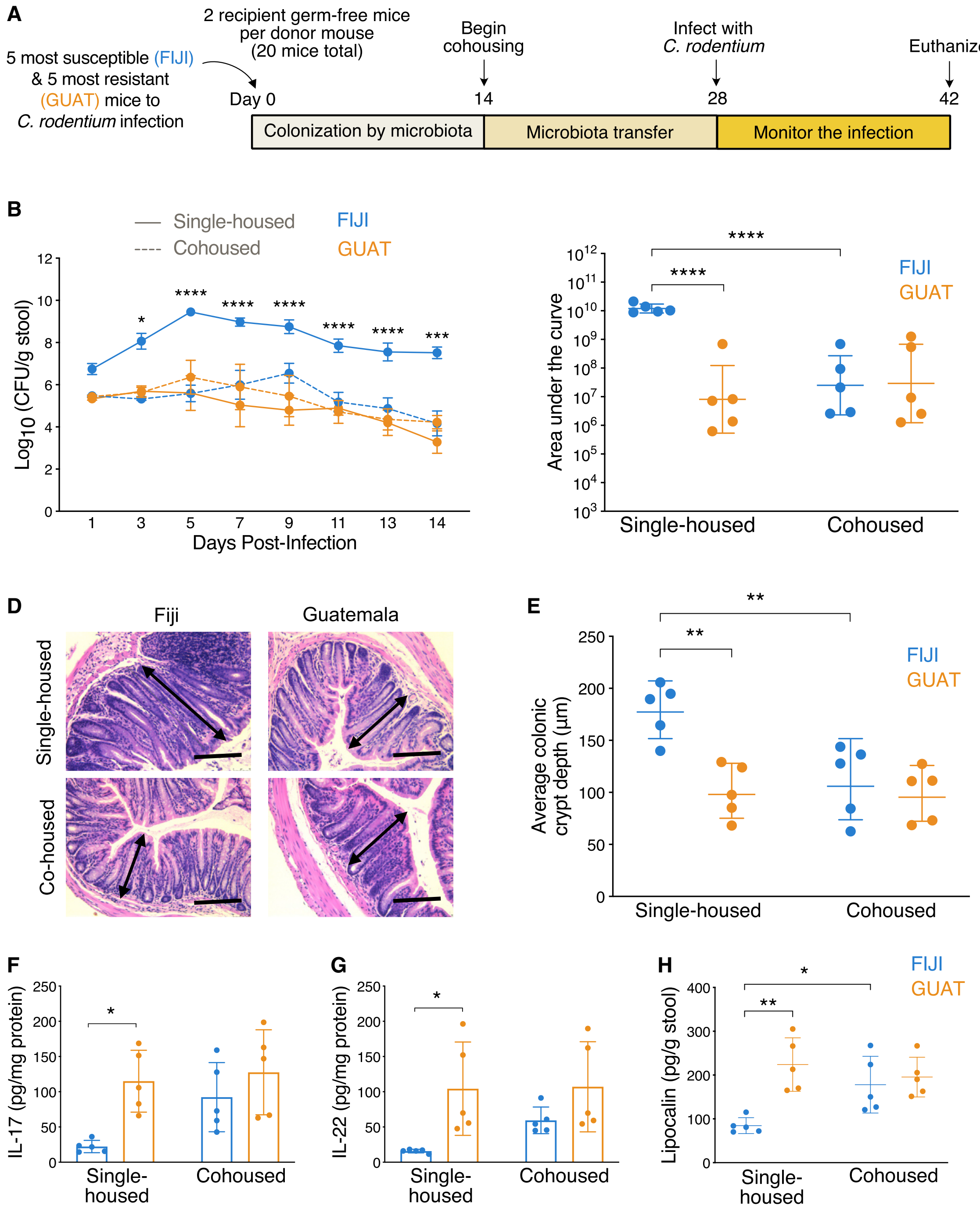

Figure 4. Resistance to *Citrobacter rodentium* infection in susceptible gnotobiotic mice is transferrable after exposure to resistant gnotobiotic mice

(A) Experimental design. After identifying the 5 most (FIJI) and 5 least (GUAT) susceptible mice to *C. rodentium* infection, we transplanted the cecal contents of those mice into germ-free mice (2 per donor, 20 mice total). Two weeks later, one mouse per donor was moved to a cage containing a mouse of the other experimental group for cohousing. Fourteen days after the beginning of the cohousing stage, all mice were infected with *C. rodentium.*

(B) Quantification of *C. rodentium* shedding in stool. Data are presented as mean ± SEM and n = 5 mice per experimental group. *p < 0.05 compared to single-housed Guatemalan mice at the same time point (repeated-measures two-way ANOVA followed by Tukey's multiple comparison test).

(C) Quantification of the area under the *C. rodentium* shedding curve.

(D) Representative images of H&E-stained colon sections 14 days after infection. Scale bar represents 100 µm.

(legend continued on next page)

shedding in the stool compared to the single-housed GUAT mice (Figure 4B). While the cohoused GUAT mice shed comparable amounts of *C. rodentium* to their single-housed counterparts, the cohoused FIJI mice excreted significantly lower levels of bacteria compared to the single-housed controls matching the levels of bacterial shedding observed in the GUAT mice (Figures 4B and 4C). Differences in pathophysiology were also evident through histological analysis of explanted colons (Figure 4D), where the average colonic crypts were significantly deeper in the single-housed FIJI mice compared to both the cohoused FIJI mice and GUAT group (Figure 4E). Furthermore, the cohoused FIJI mice secreted significantly higher quantities of IL-17 (Figure 4F) and IL-22 (Figure 4G) compared to the single-housed FIJI mice and equivalent quantities to those produced by both GUAT groups 14 days post-infection. Meanwhile, cohousing appeared to have no effect on inflammatory cytokine production for the GUAT mice (Figures 4F and 4G). No differences in the production of TNF-α (Figure S7A), IL-10 (Figure S7B), or IFN-γ (Figure S7C) were observed across donor countries or cohousing conditions. To evaluate the role of basal inflammation in this experiment, we measured LCN-2 levels in the stool on day 28, 2 weeks after cohousing, and immediately prior to inoculation with *C. rodentium*. Consistent with prior results, higher levels of LCN-2 were found in the single-housed GUAT mice compared to the single-housed FIJI mice (Figure 4H). In contrast, the cohoused FIJI mice exhibited significantly higher secretion of LCN-2 in the stool to levels comparable to both GUAT groups. Given that the cohoused GUAT mice remained resistant to the infection and the cohoused FIJI mice became less susceptible, the transfer of microbiota and subsequent increase in basal inflammation levels appeared to be sufficient in conferring resistance against *C. rodentium.*

Transfer of microbiota from resistant to susceptible mice confers resistance to infection after cohousing

We next examined the changes in the microbiome potentially responsible for this transfer of resistance to *C. rodentium* infection. We identified increases in beta diversity in the FIJI mice after 2 weeks of cohousing with GUAT mice but not their single-housed counterparts (Figures 5A and 5B; Figure S7D). Specifically, the microbiome composition of the cohoused FIJI mice started to resemble that of their GUAT counterparts. To confirm these observations, we performed a paired analysis of the ASV transfer between the pairs of cohoused mice. We identified a total of 94 ASVs that transferred from the GUAT to the FIJI mice across all pairs during cohousing (Table S4; Figure 5C). In contrast, only 41 ASVs were transferred from the FIJI to the GUAT mice (Table S4; Figure S7G). Among the ASVs transferred from the GUAT to the FIJI mice, those belonging to the *Bacteroides* genus (including *B. intestinalis*, *B. fragilis*, *B. xylanisolvens*, and *B. stercoris)*, *Akkermansia muciniphila*, and *Prevotella copri* were among those present in the FIJI guts at highest abundances after cohousing (Table S4; Figure 5C). These results perhaps suggest a role for these taxa in modulating resistance to enteric infection by *C. rodentium.*

Notably, the ASVs that originated in the GUAT microbiomes were able to colonize the FIJI mouse guts effectively, representing 28.7% ± 22.8% of the taxa identified in these mice, whereas the ASVs that originated in the FIJI mice only made up 4.8% ± 4.4% of the GUAT microbiomes 2 weeks after cohousing (Figure 5D). No statistically significant changes in alpha diversity were observed across experimental groups after 2 weeks of cohousing (Figure S7E). The transfer of microbiota from the GUAT to FIJI mice appeared to be greater than transfer in the opposite direction. We also assessed the effect of *C. rodentium* infection on the microbiomes of all mice. As was the case in the previous experiment, inoculation with this pathogen did not lead to significant changes in the microbiome composition of FIJI and GUAT mice regardless of their housing status (Figure S7F). Thus, the transfer of ASVs from the GUAT to the FIJI mice was sufficient to confer resistance to *C. rodentium* infection.

DISCUSSION

Here, we show that protection to enteric infection can be improved by the introduction of gut microbiota from donors depending on their geographic origins. We found microbiome-induced differences, according to population, in the protection against *C. rodentium* infections. More specifically, we saw increased resistance to the infection in mice that exhibited higher levels of basal inflammation after human microbiota transplantation. These effects were dynamic and could be modulated within a 2-week time period, advancing the promise of immune-modulatory microbiome-based therapies. We suspect that these effects may translate to other Th17-mediated responses, such as those involved in autoimmune disorders like rheumatoid arthritis, multiple sclerosis, or systemic lupus erythematosus, which have been linked with the microbiome (Jangi et al., 2016; López et al., 2016; Maeda et al., 2016; Scher et al., 2013). Evaluating the effects of microbiome compositions in the context of gnotobiotic mice, such as we demonstrate here, may help decouple the effects of microbiome composition from confounding factors, including population genetics, differences in diet, and disparities in access to healthcare or sanitation infrastructure. These analyses may prove important for understanding contributions of local microbiome compositions to global patterns of infectious disease and Th17-mediated autoimmune disorders (Porras and Brito, 2019; Shapira et al., 2010).

The cohousing experiment presented here highlights the malleable nature of the gut microbiome. Similar changes in microbiota composition have been reported in humans as a result of changes in diet (Gehrig et al., 2019), temporary travel (Leo et al., 2019), or permanent immigration (Vangay et al., 2018). Furthermore, the transfer of resistance to infection from GUAT to FIJI mice reinforces the potential for therapeutics that restore

(E) Quantification of average colonic crypt depth 14 days after infection.
(F and G) Quantification of the production of the inflammatory cytokines (F) IL-17 and (G) IL-22 in *ex vivo* colon culture at the end of the experiment.
(H) Assessment of LCN-2 in the stool 2 weeks after cohousing and immediately prior to inoculation with *C. rodentium.*
For (C)–(H), data are presented as mean values ± SD, with *p < 0.05, **p < 0.01, ***p < 0.001, ****p < 0.0001 for comparisons shown (two-way ANOVA followed by Tukey's multiple comparison test). See also Figure S7.

CellPress
OPEN ACCESS

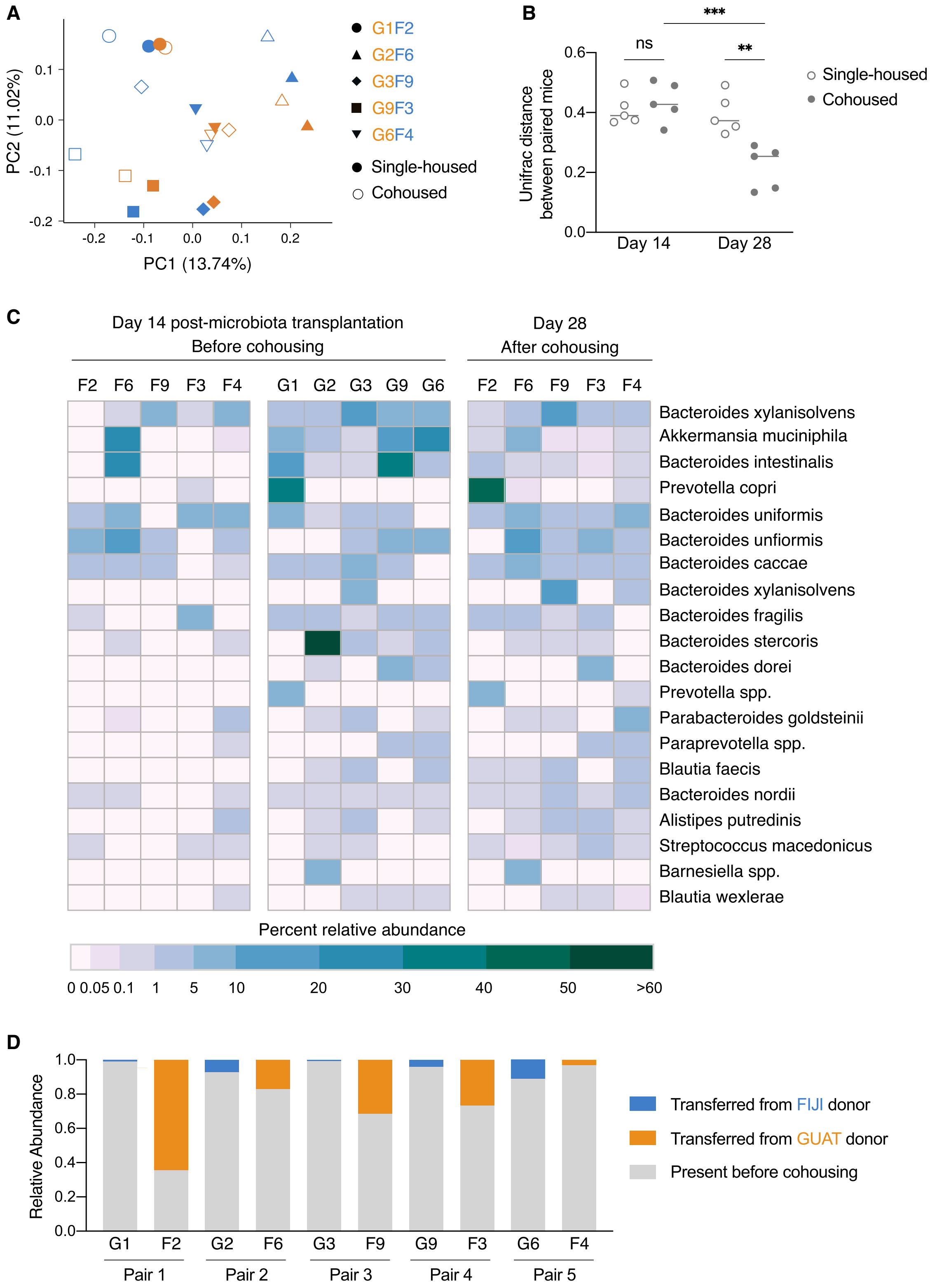

Figure 5. Transfer of microbiota from resistant to susceptible mice confers resistance to infection after cohousing

(A) PCoA of unweighted UniFrac distances for the gut microbiomes of the mice on day 28 of the experiment, 2 weeks after cohousing. Labels for the paired mice are found next to each other on the figure legend.

(legend continued on next page)

healthy functions to the host through the modification of the gut microbiome. This is consistent with both mouse (Rosshart et al., 2017; Smith et al., 2019; Villarino et al., 2016; Willing et al., 2011) and human microbiota transplantation studies in the context of resistance to infection (AlQahtani et al., 2020; Cheng et al., 2019, 2020; Ianiro et al., 2018; Jiang et al., 2017; Krajicek et al., 2019; Shin et al., 2019). These results support the hypothesis that geographic-level differences in gut microbiome composition may influence health outcomes. Our data supports a causal role for the microbiota irrespective of direct impacts of population genetics, health infrastructure, and other confounding factors. Nonetheless, we caution against interpreting these results as evidence of the relative benefits or side effects of any specific populations' microbiome composition over another for various reasons. First, these experiments measure a single health outcome, susceptibility to *C. rodentium* and *L. monocytogenes* infection. Additionally, microbiota or microbiome compositions identified as deleterious for one outcome may provide a health benefit in another context or vice versa (Gilbert and Lynch, 2019). Finally, the interpretation of gnotobiotic mouse studies must consider the possibility that differences between cohorts arise due to the underlying colonization potential of the microbiomes being tested. Our results cannot exclude this explanation, as the FIJI mice experienced lower colonization by donor-associated microbiota overall. Mouse studies, including ours, provide initial validation for hypotheses but require follow-up experiments in human populations.

Our work also emphasizes the importance of study design in drawing conclusions from necessarily small-scale germ-free or gnotobiotic mouse experiments. Gnotobiotic mouse experiments require additional care to ensure control over the gut microenvironment and therefore are not amenable to a large number of donors. A recent meta-analysis of this type of experiments (Walter et al., 2020) found that most studies use a small (one to five) number of donors, whose microbiomes are then transplanted into a larger number of mice. Careful choice of multiple representative donors is therefore critical in extrapolating results obtained with small sample sizes to entire disease cohorts or geographic populations. A strength of our approach is our utilization of 30 individuals as donors, an unusually large number of donors, across several control and experimental conditions. The inherent limitations of germ-free mouse work can be partially mitigated by the use of more donors and accounting of colonization efficacy.

Overall, this study highlights the need to expand microbiome studies to account for the greater diversity in microbiome compositions globally not only in cohort-based studies (Mejía-León et al., 2014; Monaco et al., 2016; Subramanian et al., 2014; Zhong et al., 2019) but also in gnotobiotic animal experiments, which serve as a predominant tool for examining causal linkages between the microbiome and disease. On one hand, these studies may reveal population-level differences that can contribute to our understanding of the mechanisms by which the microbiome affects immunity and disease and which may contribute to geographic differences in incidence. At the same time, comparative studies may also uncover universal principles that more broadly capture mechanistic links between the microbiome and disease. Increasing the participation and inclusion of diverse populations around the world is crucial for the advancement of biomedicine. The historically poor representation of women and racial/ethnic minorities in clinical trials performed in the United States (Coakley et al., 2012; Oh et al., 2015) has resulted in the under-appreciation of sex-based or ethnicity/race-based differences in disease risk and therapeutic responses (Anderson, 2005; Dirks et al., 2004; Phan et al., 2009; Rotger et al., 2006; Zopf et al., 2008). Intentional study designs that consider the vast differences in microbiome composition across global populations will impact the development of future strategies to prevent or treat disease and inform their expected efficacy across populations (Brooks et al., 2018; Gehrig et al., 2019; Klatt et al., 2017; Mosquera et al., 2019; Panigrahi et al., 2017; Rekdal et al., 2019). This is crucial for the development of microbiome-based solutions to global health challenges.

STAR★METHODS

Detailed methods are provided in the online version of this paper and include the following:

- KEY RESOURCES TABLE
- RESOURCE AVAILABILITY
  - Lead contact
  - Materials availability
  - Data and code availability
- EXPERIMENTAL MODEL AND SUBJECT DETAILS
  - Human stool donors
  - Mouse strain and husbandry
  - Bacterial strains
- METHOD DETAILS
  - Preparation of human stool slurries
  - C. rodentium infection model
  - Monitoring C. rodentium shedding
  - Listeria Monocytogenes Infection Model
  - Measurement of L. monocytogenes burden in the liver and spleen
  - Cohousing experiment
  - Histological and immunofluorescent characterization of explanted colons
  - Quantification of LCN-2 and calprotectin in mouse stool
  - Quantification of inflammatory cytokine production *ex vivo*
  - 16S rRNA gene sequencing

(B) UniFrac distance between paired mice prior to (day 14) and after (day 28) the cohousing period. Horizontal line represents the mean, with **$p < 0.01$ and ***$p < 0.001$ for comparisons shown (two-way ANOVA followed by Tukey's multiple comparison test).
(C) Heatmap of the percent relative abundances of the top 20 most abundant taxa transferred from the GUAT mice to their FIJI cohousing partners after cohousing. The mice are presented according to their corresponding cohousing pair.
(D) Quantification of the relative abundances of the ASVs originally present in the mice prior to cohousing and transferred from their cohousing partner. See also Figure S7 and Table S4.

- QUANTIFICATION AND STATISTICAL ANALYSIS
  - Analysis of 16S rRNA gene sequencing
  - Human donor selection and comparison to their respective populations
  - Statistical analysis

SUPPLEMENTAL INFORMATION

Supplemental information can be found online at https://doi.org/10.1016/j.celrep.2021.109457.

ACKNOWLEDGMENTS

This work was partially supported by the Gates Foundation (OPP1161064). Additionally, A.M.P. was supported throughout this work by funding from Cornell University's Presidential Postdoctoral Fellowship program.

AUTHOR CONTRIBUTIONS

Conceptualization, A.M.P., and I.L.B.; resources, G.M.B., and N.S.; methodology, A.M.P., Q.S., and I.L.B.; validation, A.M.P. and Q.S.; investigation, A.M.P., R.C., and Q.S.; formal analysis, data curation, and visualization, A.M.P. and H.Z.; writing – original draft, A.M.P. and I.L.B.; writing – review & editing, all authors; supervision, I.L.B.; project administration, A.M.P., Q.S., and I.L.B.; funding acquisition: I.L.B.

DECLARATION OF INTERESTS

The authors declare no competing interests.

INCLUSION AND DIVERSITY

We worked to ensure gender balance in the recruitment of human subjects. We worked to ensure ethnic or other types of diversity in the recruitment of human subjects. We worked to ensure that the study questionnaires were prepared in an inclusive way. One or more of the authors of this paper self-identifies as an underrepresented ethnic minority in science. The author list of this paper includes contributors from the location where the research was conducted who participated in the data collection, design, analysis, and/or interpretation of the work.

Received: January 26, 2021
Revised: May 26, 2021
Accepted: July 7, 2021
Published: July 27, 2021

REFERENCES

Almeida, A., Mitchell, A.L., Boland, M., Forster, S.C., Gloor, G.B., Tarkowska, A., Lawley, T.D., and Finn, R.D. (2019). A new genomic blueprint of the human gut microbiota. Nature *568*, 499–504.

AlQahtani, H., Baloch, S., and Tabb, D. (2020). Treatment of Recurrent *Clostridium difficile* Infection in an Immunocompromised Patient with Severe Neutropenia Not Responding to Standard Therapy. Case Rep. Infect. Dis. *2020*, 3089023.

Anderson, G.D. (2005). Sex and racial differences in pharmacological response: where is the evidence? Pharmacogenetics, pharmacokinetics, and pharmacodynamics. J. Womens Health (Larchmt.) *14*, 19–29.

Apprill, A., McNally, S., Parsons, R., and Weber, L. (2015). Minor revision to V4 region SSU rRNA 806R gene primer greatly increases detection of SAR11 bacterioplankton. Aquat. Microb. Ecol. *75*, 129–137.

Atarashi, K., Tanoue, T., Ando, M., Kamada, N., Nagano, Y., Narushima, S., Suda, W., Imaoka, A., Setoyama, H., Nagamori, T., et al. (2015). Th17 Cell Induction by Adhesion of Microbes to Intestinal Epithelial Cells. Cell *163*, 367–380.

Bailey, M.T., Dowd, S.E., Parry, N.M.A., Galley, J.D., Schauer, D.B., and Lyte, M. (2010). Stressor exposure disrupts commensal microbial populations in the intestines and leads to increased colonization by Citrobacter rodentium. Infect. Immun. *78*, 1509–1519.

Basu, R., O'Quinn, D.B., Silberger, D.J., Schoeb, T.R., Fouser, L., Ouyang, W., Hatton, R.D., and Weaver, C.T. (2012). Th22 cells are an important source of IL-22 for host protection against enteropathogenic bacteria. Immunity *37*, 1061–1075.

Baxter, N.T., Zackular, J.P., Chen, G.Y., and Schloss, P.D. (2014). Structure of the gut microbiome following colonization with human feces determines colonic tumor burden. Microbiome *2*, 20.

Becattini, S., Littmann, E.R., Carter, R.A., Kim, S.G., Morjaria, S.M., Ling, L., Gyaltshen, Y., Fontana, E., Taur, Y., Leiner, I.M., and Pamer, E.G. (2017). Commensal microbes provide first line defense against *Listeria monocytogenes* infection. J. Exp. Med. *214*, 1973–1989.

Berer, K., Gerdes, L.A., Cekanaviciute, E., Jia, X., Xiao, L., Xia, Z., Liu, C., Klotz, L., Stauffer, U., Baranzini, S.E., et al. (2017). Gut microbiota from multiple sclerosis patients enables spontaneous autoimmune encephalomyelitis in mice. Proc. Natl. Acad. Sci. USA *114*, 10719–10724.

Bolyen, E., Rideout, J.R., Dillon, M.R., Bokulich, N.A., Abnet, C.C., Al-Ghalith, G.A., Alexander, H., Alm, E.J., Arumugam, M., Asnicar, F., et al. (2019). Reproducible, interactive, scalable and extensible microbiome data science using QIIME 2. Nat. Biotechnol. *37*, 852–857.

Brewster, R., Tamburini, F.B., Asiimwe, E., Oduaran, O., Hazelhurst, S., and Bhatt, A.S. (2019). Surveying Gut Microbiome Research in Africans: Toward Improved Diversity and Representation. Trends Microbiol. *27*, 824–835.

Brito, I.L., Yilmaz, S., Huang, K., Xu, L., Jupiter, S.D., Jenkins, A.P., Naisilisili, W., Tamminen, M., Smillie, C.S., Wortman, J.R., et al. (2016). Mobile genes in the human microbiome are structured from global to individual scales. Nature *535*, 435–439.

Britton, G.J., Contijoch, E.J., Mogno, I., Vennaro, O.H., Llewellyn, S.R., Ng, R., Li, Z., Mortha, A., Merad, M., Das, A., et al. (2019). Microbiotas from Humans with Inflammatory Bowel Disease Alter the Balance of Gut Th17 and RORγt^{+} Regulatory T Cells and Exacerbate Colitis in Mice. Immunity *50*, 212–224.e4.

Brooks, A.W., Priya, S., Blekhman, R., and Bordenstein, S.R. (2018). Gut microbiota diversity across ethnicities in the United States. PLoS Biol. *16*, e2006842.

Brown, E.M., Ke, X., Hitchcock, D., Jeanfavre, S., Avila-Pacheco, J., Nakata, T., Arthur, T.D., Fornelos, N., Heim, C., Franzosa, E.A., et al. (2019). Bacteroides-Derived Sphingolipids Are Critical for Maintaining Intestinal Homeostasis and Symbiosis. Cell Host Microbe *25*, 668–680.

Caballero, S., and Pamer, E.G. (2015). Microbiota-Mediated Inflammation and Antimicrobial Defense in the Intestine. Annu. Rev. Immunol. *33*, 227–256.

Callahan, B.J., McMurdie, P.J., Rosen, M.J., Han, A.W., Johnson, A.J.A., and Holmes, S.P. (2016). DADA2: High-resolution sample inference from Illumina amplicon data. Nat. Methods *13*, 581–583.

Caporaso, J.G., Kuczynski, J., Stombaugh, J., Bittinger, K., Bushman, F.D., Costello, E.K., Fierer, N., Peña, A.G., Goodrich, J.K., Gordon, J.I., et al. (2010). QIIME allows analysis of high-throughput community sequencing data. Nat. Methods *7*, 335–336.

Caporaso, J.G., Lauber, C.L., Walters, W.A., Berg-Lyons, D., Lozupone, C.A., Turnbaugh, P.J., Fierer, N., and Knight, R. (2011). Global patterns of 16S rRNA diversity at a depth of millions of sequences per sample. Proc. Natl. Acad. Sci. USA *108* (*Suppl 1*), 4516–4522.

Chassaing, B., Srinivasan, G., Delgado, M.A., Young, A.N., Gewirtz, A.T., and Vijay-Kumar, M. (2012). Fecal lipocalin 2, a sensitive and broadly dynamic non-invasive biomarker for intestinal inflammation. PLoS ONE *7*, e44328.

Chen, J., Waddell, A., Lin, Y.-D., and Cantorna, M.T. (2015). Dysbiosis caused by vitamin D receptor deficiency confers colonization resistance to Citrobacter rodentium through modulation of innate lymphoid cells. Mucosal Immunol. *8*, 618–626.

Cheng, Y.-W., Phelps, E., Ganapini, V., Khan, N., Ouyang, F., Xu, H., Khanna, S., Tariq, R., Friedman-Moraco, R.J., Woodworth, M.H., et al. (2019). Fecal

microbiota transplantation for the treatment of recurrent and severe *Clostridium difficile* infection in solid organ transplant recipients: A multicenter experience. Am. J. Transplant. *19*, 501–511.

Cheng, Y.-W., Phelps, E., Nemes, S., Rogers, N., Sagi, S., Bohm, M., El-Halabi, M., Allegretti, J.R., Kassam, Z., Xu, H., and Fischer, M. (2020). Fecal Microbiota Transplant Decreases Mortality in Patients with Refractory Severe or Fulminant Clostridioides difficile Infection. Clin. Gastroenterol. Hepatol. *18*, 2234–2243.

Chiu, C.-C., Ching, Y.-H., Li, Y.-P., Liu, J.-Y., Huang, Y.-T., Huang, Y.-W., Yang, S.-S., Huang, W.-C., and Chuang, H.-L. (2017). Nonalcoholic Fatty Liver Disease Is Exacerbated in High-Fat Diet-Fed Gnotobiotic Mice by Colonization with the Gut Microbiota from Patients with Nonalcoholic Steatohepatitis. Nutrients *9*, 1220.

Coakley, M., Fadiran, E.O., Parrish, L.J., Griffith, R.A., Weiss, E., and Carter, C. (2012). Dialogues on diversifying clinical trials: successful strategies for engaging women and minorities in clinical trials. J. Womens Health (Larchmt.) *21*, 713–716.

Collins, J.W., Keeney, K.M., Crepin, V.F., Rathinam, V.A.K., Fitzgerald, K.A., Finlay, B.B., and Frankel, G. (2014). Citrobacter rodentium: infection, inflammation and the microbiota. Nat. Rev. Microbiol. *12*, 612–623.

Crepin, V.F., Collins, J.W., Habibzay, M., and Frankel, G. (2016). Citrobacter rodentium mouse model of bacterial infection. Nat. Protoc. *11*, 1851–1876.

De Filippo, C., Cavalieri, D., Di Paola, M., Ramazzotti, M., Poullet, J.B., Massart, S., Collini, S., Pieraccini, G., and Lionetti, P. (2010). Impact of diet in shaping gut microbiota revealed by a comparative study in children from Europe and rural Africa. Proc. Natl. Acad. Sci. USA *107*, 14691–14696.

De Palma, G., Lynch, M.D.J., Lu, J., Dang, V.T., Deng, Y., Jury, J., Umeh, G., Miranda, P.M., Pigrau Pastor, M., Sidani, S., et al. (2017). Transplantation of fecal microbiota from patients with irritable bowel syndrome alters gut function and behavior in recipient mice. Sci. Transl. Med. *9*, eaaf6397.

Dirks, N.L., Huth, B., Yates, C.R., and Meibohm, B. (2004). Pharmacokinetics of immunosuppressants: a perspective on ethnic differences. Int. J. Clin. Pharmacol. Ther. *42*, 701–718.

Fouladi, F., Glenny, E.M., Bulik-Sullivan, E.C., Tsilimigras, M.C.B., Sioda, M., Thomas, S.A., Wang, Y., Djukic, Z., Tang, Q., Tarantino, L.M., et al. (2020). Sequence variant analysis reveals poor correlations in microbial taxonomic abundance between humans and mice after gnotobiotic transfer. ISME J. *14*, 1809–1820.

GBD 2016 Diarrheal Disease Collaborators (2018). Estimates of the global, regional, and national morbidity, mortality, and aetiologies of diarrhoea in 195 countries: a systematic analysis for the Global Burden of Disease Study 2016. Lancet Infect. Dis. *18*, 1211–1228.

Gehrig, J.L., Venkatesh, S., Chang, H.-W., Hibberd, M.C., Kung, V.L., Cheng, J., Chen, R.Y., Subramanian, S., Cowardin, C.A., Meier, M.F., et al. (2019). Effects of microbiota-directed foods in gnotobiotic animals and undernourished children. Science *365*, eaau4732.

Gilbert, J.A., and Lynch, S.V. (2019). Community ecology as a framework for human microbiome research. Nat. Med. *25*, 884–889.

Gupta, V.K., Paul, S., and Dutta, C. (2017). Geography, Ethnicity or Subsistence-Specific Variations in Human Microbiome Composition and Diversity. Front. Microbiol. *8*, 1162.

He, Y., Wu, W., Zheng, H.-M., Li, P., McDonald, D., Sheng, H.-F., Chen, M.-X., Chen, Z.-H., Ji, G.-Y., Zheng, Z.-D.-X., et al. (2018). Regional variation limits applications of healthy gut microbiome reference ranges and disease models. Nat. Med. *24*, 1532–1535.

Hickey, C.A., Kuhn, K.A., Donermeyer, D.L., Porter, N.T., Jin, C., Cameron, E.A., Jung, H., Kaiko, G.E., Wegorzewska, M., Malvin, N.P., et al. (2015). Colitogenic Bacteroides thetaiotaomicron antigens access host immune cells in a sulfatase-dependent manner via outer membrane vesicles. Cell Host Microbe *17*, 672–680.

Hoffmann, C., Hill, D.A., Minkah, N., Kirn, T., Troy, A., Artis, D., and Bushman, F. (2009). Community-wide response of the gut microbiota to enteropathogenic Citrobacter rodentium infection revealed by deep sequencing. Infect. Immun. *77*, 4668–4678.

Hsieh, C.S., Macatonia, S.E., Tripp, C.S., Wolf, S.F., O'Garra, A., and Murphy, K.M. (1993). Development of TH1 CD4+ T cells through IL-12 produced by Listeria-induced macrophages. Science *260*, 547–549.

Hu, H.-J., Park, S.-G., Jang, H.B., Choi, M.K., Park, K.-H., Kang, J.H., Park, S.I., Lee, H.-J., and Cho, S.-H. (2015). Obesity Alters the Microbial Community Profile in Korean Adolescents. PLoS ONE *10*, e0134333.

Ianiro, G., Masucci, L., Quaranta, G., Simonelli, C., Lopetuso, L.R., Sanguinetti, M., Gasbarrini, A., and Cammarota, G. (2018). Randomised clinical trial: faecal microbiota transplantation by colonoscopy plus vancomycin for the treatment of severe refractory *Clostridium difficile* infection-single versus multiple infusions. Aliment. Pharmacol. Ther. *48*, 152–159.

Ivanov, I.I., Frutos, R. de L., Manel, N., Yoshinaga, K., Rifkin, D.B., Sartor, R.B., Finlay, B.B., and Littman, D.R. (2008). Specific microbiota direct the differentiation of IL-17-producing T-helper cells in the mucosa of the small intestine. Cell Host Microbe *4*, 337–349.

Ivanov, I.I., Atarashi, K., Manel, N., Brodie, E.L., Shima, T., Karaoz, U., Wei, D., Goldfarb, K.C., Santee, C.A., Lynch, S.V., et al. (2009). Induction of intestinal Th17 cells by segmented filamentous bacteria. Cell *139*, 485–498.

Jacobson, A., Lam, L., Rajendram, M., Tamburini, F., Honeycutt, J., Pham, T., Van Treuren, W., Pruss, K., Stabler, S.R., Lugo, K., et al. (2018). A Gut Commensal-Produced Metabolite Mediates Colonization Resistance to Salmonella Infection. Cell Host Microbe *24*, 296–307.e7.

Jangi, S., Gandhi, R., Cox, L.M., Li, N., von Glehn, F., Yan, R., Patel, B., Mazzola, M.A., Liu, S., Glanz, B.L., et al. (2016). Alterations of the human gut microbiome in multiple sclerosis. Nat. Commun. *7*, 12015.

Jiang, Z.D., Ajami, N.J., Petrosino, J.F., Jun, G., Hanis, C.L., Shah, M., Hochman, L., Ankoma-Sey, V., DuPont, A.W., Wong, M.C., et al. (2017). Randomised clinical trial: faecal microbiota transplantation for recurrent *Clostridum difficile* infection - fresh, or frozen, or lyophilised microbiota from a small pool of healthy donors delivered by colonoscopy. Aliment. Pharmacol. Ther. *45*, 899–908.

Klatt, N.R., Cheu, R., Birse, K., Zevin, A.S., Perner, M., Noël-romas, L., Grobler, A., Westmacott, G., Xie, I.Y., Butler, J., et al. (2017). Vaginal bacteria modify HIV tenofovor microbicide efficacin African women. Science *945*, 938–945.

Koeth, R.A., Wang, Z., Levison, B.S., Buffa, J.A., Org, E., Sheehy, B.T., Britt, E.B., Fu, X., Wu, Y., Li, L., et al. (2013). Intestinal microbiota metabolism of L-carnitine, a nutrient in red meat, promotes atherosclerosis. Nat. Med. *19*, 576–585.

Kovatcheva-Datchary, P., Nilsson, A., Akrami, R., Lee, Y.S., De Vadder, F., Arora, T., Hallen, A., Martens, E., Björck, I., and Bäckhed, F. (2015). Dietary Fiber-Induced Improvement in Glucose Metabolism Is Associated with Increased Abundance of Prevotella. Cell Metab. *22*, 971–982.

Krajicek, E., Bohm, M., Sagi, S., and Fischer, M. (2019). Fulminant *Clostridium difficile* Infection Cured by Fecal Microbiota Transplantation in a Bone Marrow Transplant Recipient With Critical Neutropenia. ACG Case Rep. J. *6*, e00198.

Lee, C.H., Steiner, T., Petrof, E.O., Smieja, M., Roscoe, D., Nematallah, A., Weese, J.S., Collins, S., Moayyedi, P., Crowther, M., et al. (2016). Frozen vs fresh fecal microbiota transplantation and clinical resolution of diarrhea in patients with recurrent clostridium difficile infection a randomized clinical trial. JAMA -. JAMA *315*, 142–149.

Leo, S., Lazarevic, V., Gaïa, N., Estellat, C., Girard, M., Matheron, S., Armand-Lefèvre, L., Andremont, A., Schrenzel, J., and Ruppé, E. (2019). The intestinal microbiota predisposes to traveler's diarrhea and to the carriage of multidrug-resistant Enterobacteriaceae after traveling to tropical regions. Gut Microbes *10*, 631–641.

Libertucci, J., and Young, V.B. (2019). The role of the microbiota in infectious diseases. Nat. Microbiol. *4*, 35–45.

Lin, A., Bik, E.M., Costello, E.K., Dethlefsen, L., Haque, R., Relman, D.A., and Singh, U. (2013). Distinct distal gut microbiome diversity and composition in healthy children from Bangladesh and the United States. PLoS ONE *8*, e53838.

CellPress
OPEN ACCESS

López, P., de Paz, B., Rodríguez-Carrio, J., Hevia, A., Sánchez, B., Margolles, A., and Suárez, A. (2016). Th17 responses and natural IgM antibodies are related to gut microbiota composition in systemic lupus erythematosus patients. Sci. Rep. *6*, 24072.

Maeda, Y., Kurakawa, T., Umemoto, E., Motooka, D., Ito, Y., Gotoh, K., Hirota, K., Matsushita, M., Furuta, Y., Narazaki, M., et al. (2016). Dysbiosis Contributes to Arthritis Development via Activation of Autoreactive T Cells in the Intestine. Arthritis Rheumatol. *68*, 2646–2661.

Magne, F., O'Ryan, M.L., Vidal, R., and Farfan, M. (2016). The human gut microbiome of Latin America populations: a landscape to be discovered. Curr. Opin. Infect. Dis. *29*, 528–537.

Mandal, S., Van Treuren, W., White, R.A., Eggesbø, M., Knight, R., and Peddada, S.D. (2015). Analysis of composition of microbiomes: a novel method for studying microbial composition. Microb. Ecol. Health Dis. *26*, 27663.

Mandeville, K.L., Krabshuis, J., Ladep, N.G., Mulder, C.J.J., Quigley, E.M.M., and Khan, S.A. (2009). Gastroenterology in developing countries: issues and advances. World J. Gastroenterol. *15*, 2839–2854.

Martínez, I., Stegen, J.C., Maldonado-Gómez, M.X., Eren, A.M., Siba, P.M., Greenhill, A.R., and Walter, J. (2015). The gut microbiota of rural papua new guineans: composition, diversity patterns, and ecological processes. Cell Rep. *11*, 527–538.

McKenney, P.T., and Pamer, E.G. (2015). From Hype to Hope: The Gut Microbiota in Enteric Infectious Disease. Cell *163*, 1326–1332.

McMurdie, P.J., and Holmes, S. (2013). phyloseq: an R package for reproducible interactive analysis and graphics of microbiome census data. PLoS ONE *8*, e61217.

Mejía-León, M.E., Petrosino, J.F., Ajami, N.J., Domínguez-Bello, M.G., and de la Barca, A.M.C. (2014). Fecal microbiota imbalance in Mexican children with type 1 diabetes. Sci. Rep. *4*, 3814.

Midani, F.S., Weil, A.A., Chowdhury, F., Begum, Y.A., Khan, A.I., Debela, M.D., Durand, H.K., Reese, A.T., Nimmagadda, S.N., Silverman, J.D., et al. (2018). Human Gut Microbiota Predicts Susceptibility to Vibrio cholerae Infection. J. Infect. Dis. *218*, 645–653.

Monaco, C.L., Gootenberg, D.B., Zhao, G., Handley, S.A., Ghebremichael, M.S., Lim, E.S., Lankowski, A., Baldridge, M.T., Wilen, C.B., Flagg, M., et al. (2016). Altered Virome and Bacterial Microbiome in Human Immunodeficiency Virus-Associated Acquired Immunodeficiency Syndrome. Cell Host Microbe *19*, 311–322.

Morton, E.R., Lynch, J., Froment, A., Lafosse, S., Heyer, E., Przeworski, M., Blekhman, R., and Ségurel, L. (2015). Variation in Rural African Gut Microbiota Is Strongly Correlated with Colonization by Entamoeba and Subsistence. PLoS Genet. *11*, e1005658.

Mosquera, M.J., Kim, S., Zhou, H., Jing, T.T., Luna, M., Guss, J.D., Reddy, P., Lai, K., Leifer, C.A., Brito, I.L., et al. (2019). Immunomodulatory nanogels overcome restricted immunity in a murine model of gut microbiome-mediated metabolic syndrome. Sci. Adv. *5*, eaav9788.

Mullineaux-Sanders, C., Collins, J.W., Ruano-Gallego, D., Levy, M., Pevsner-Fischer, M., Glegola-Madejska, I.T., Sågfors, A.M., Wong, J.L.C., Elinav, E., Crepin, V.F., and Frankel, G. (2017). Citrobacter rodentium Relies on Commensals for Colonization of the Colonic Mucosa. Cell Rep. *21*, 3381–3389.

Nayfach, S., Shi, Z.J., Seshadri, R., Pollard, K.S., and Kyrpides, N.C. (2019). New insights from uncultivated genomes of the global human gut microbiome. Nature *568*, 505–510.

Ni, J., Shen, T.D., Chen, E.Z., Bittinger, K., Bailey, A., Roggiani, M., Sirota-Madi, A., Friedman, E.S., Chau, L., Lin, A., et al. (2017). A role for bacterial urease in gut dysbiosis and Crohn's disease. Sci. Transl. Med. *9*, 9.

Oh, S.S., Galanter, J., Thakur, N., Pino-Yanes, M., Barcelo, N.E., White, M.J., de Bruin, D.M., Greenblatt, R.M., Bibbins-Domingo, K., Wu, A.H.B., et al. (2015). Diversity in Clinical and Biomedical Research: A Promise Yet to Be Fulfilled. PLoS Med. *12*, e1001918.

Olesen, S.W., Panchal, P., Chen, J., Budree, S., and Osman, M. (2020). Global disparities in faecal microbiota transplantation research. Lancet Gastroenterol. Hepatol. *5*, 241.

Panigrahi, P., Parida, S., Nanda, N.C., Satpathy, R., Pradhan, L., Chandel, D.S., Baccaglini, L., Mohapatra, A., Mohapatra, S.S., Misra, P.R., et al. (2017). A randomized synbiotic trial to prevent sepsis among infants in rural India. Nature *548*, 407–412.

Parada, A.E., Needham, D.M., and Fuhrman, J.A. (2016). Every base matters: assessing small subunit rRNA primers for marine microbiomes with mock communities, time series and global field samples. Environ. Microbiol. *18*, 1403–1414.

Pasolli, E., Asnicar, F., Manara, S., Zolfo, M., Karcher, N., Armanini, F., Beghini, F., Manghi, P., Tett, A., Ghensi, P., et al. (2019). Extensive Unexplored Human Microbiome Diversity Revealed by Over 150,000 Genomes from Metagenomes Spanning Age, Geography, and Lifestyle. Cell *176*, 649–662.e20.

Phan, V.H., Moore, M.M., McLachlan, A.J., Piquette-Miller, M., Xu, H., and Clarke, S.J. (2009). Ethnic differences in drug metabolism and toxicity from chemotherapy. Expert Opin. Drug Metab. Toxicol. *5*, 243–257.

Plovier, H., Everard, A., Druart, C., Depommier, C., Van Hul, M., Geurts, L., Chilloux, J., Ottman, N., Duparc, T., Lichtenstein, L., et al. (2017). A purified membrane protein from Akkermansia muciniphila or the pasteurized bacterium improves metabolism in obese and diabetic mice. Nat. Med. *23*, 107–113.

Png, C.W., Lindén, S.K., Gilshenan, K.S., Zoetendal, E.G., McSweeney, C.S., Sly, L.I., McGuckin, M.A., and Florin, T.H.J. (2010). Mucolytic bacteria with increased prevalence in IBD mucosa augment in vitro utilization of mucin by other bacteria. Am. J. Gastroenterol. *105*, 2420–2428.

Porras, A.M., and Brito, I.L. (2019). The internationalization of human microbiome research. Curr. Opin. Microbiol. *50*, 50–55.

Rekdal, V.M., Bess, E.N., Bisanz, J.E., Turnbaugh, P.J., and Balskus, E.P. (2019). Discovery and inhibition of an interspecies gut bacterial pathway for Levodopa metabolism. Science *364*, 1055.

Ridaura, V.K., Faith, J.J., Rey, F.E., Cheng, J., Duncan, A.E., Kau, A.L., Griffin, N.W., Lombard, V., Henrissat, B., Bain, J.R., et al. (2013). Gut Microbiota from Twins Discordant for Obesity Modulate Metabolism in Mice. Science *341*, 1241214–1241214.

Rosshart, S.P., Vassallo, B.G., Angeletti, D., Hutchinson, D.S., Morgan, A.P., Takeda, K., Hickman, H.D., McCulloch, J.A., Badger, J.H., Ajami, N.J., et al. (2017). Wild Mouse Gut Microbiota Promotes Host Fitness and Improves Disease Resistance. Cell *171*, 1015–1028.e13.

Rotger, M., Csajka, C., and Telenti, A. (2006). Genetic, ethnic, and gender differences in the pharmacokinetics of antiretroviral agents. Curr. HIV/AIDS Rep. *3*, 118–125.

Rubel, M.A., Abbas, A., Taylor, L.J., Connell, A., Tanes, C., Bittinger, K., Ndze, V.N., Fonsah, J.Y., Ngwang, E., Essiane, A., et al. (2020). Lifestyle and the presence of helminths is associated with gut microbiome composition in Cameroonians. Genome Biol. *21*, 122.

Rudd, B.D., Venturi, V., Li, G., Samadder, P., Ertelt, J.M., Way, S.S., Davenport, M.P., and Nikolich-Žugich, J. (2011). Nonrandom attrition of the naive CD8+ T-cell pool with aging governed by T-cell receptor:pMHC interactions. Proc. Natl. Acad. Sci. USA *108*, 13694–13699.

Saitoh, S., Noda, S., Aiba, Y., Takagi, A., Sakamoto, M., Benno, Y., and Koga, Y. (2002). Bacteroides ovatus as the predominant commensal intestinal microbe causing a systemic antibody response in inflammatory bowel disease. Clin. Diagn. Lab. Immunol. *9*, 54–59.

Scher, J.U., Sczesnak, A., Longman, R.S., Segata, N., Ubeda, C., Bielski, C., Rostron, T., Cerundolo, V., Pamer, E.G., Abramson, S.B., et al. (2013). Expansion of intestinal Prevotella copri correlates with enhanced susceptibility to arthritis. eLife *2*, e01202.

Shapira, Y., Agmon-Levin, N., and Shoenfeld, Y. (2010). Defining and analyzing geoepidemiology and human autoimmunity. J. Autoimmun. *34*, J168–J177.

Shin, J.H., Chaplin, A.S., Hays, R.A., Kolling, G.L., Vance, S., Guerrant, R.L., Archbald-Pannone, L., and Warren, C.A. (2019). Outcomes of a Multidisciplinary Clinic in Evaluating Recurrent *Clostridioides difficile* Infection Patients for Fecal Microbiota Transplant: A Retrospective Cohort Analysis. J. Clin. Med. *8*, 1036.

Silberger, D.J., Zindl, C.L., and Weaver, C.T. (2017). Citrobacter rodentium: a model enteropathogen for understanding the interplay of innate and adaptive components of type 3 immunity. Mucosal Immunol. *10*, 1108–1117.

Smith, N.L., Patel, R.K., Reynaldi, A., Grenier, J.K., Wang, J., Watson, N.B., Nzingha, K., Yee Mon, K.J., Peng, S.A., Grimson, A., et al. (2018). Developmental Origin Governs $CD8^+$ T Cell Fate Decisions during Infection. Cell *174*, 117–130.e14.

Smith, A.D., Foss, E.D., Zhang, I., Hastie, J.L., Giordano, N.P., Gasparyan, L., VinhNguyen, L.P., Schubert, A.M., Prasad, D., McMichael, H.L., et al. (2019). Microbiota of MR1 deficient mice confer resistance against Clostridium difficile infection. PLoS ONE *14*, e0223025.

Sonnenburg, E.D., and Sonnenburg, J.L. (2019). The ancestral and industrialized gut microbiota and implications for human health. Nat. Rev. Microbiol. *17*, 383–390.

Subramanian, S., Huq, S., Yatsunenko, T., Haque, R., Mahfuz, M., Alam, M.A., Benezra, A., DeStefano, J., Meier, M.F., Muegge, B.D., et al. (2014). Persistent gut microbiota immaturity in malnourished Bangladeshi children. Nature *510*, 417–421.

Turnbaugh, P.J., Ley, R.E., Mahowald, M.A., Magrini, V., Mardis, E.R., and Gordon, J.I. (2006). An obesity-associated gut microbiome with increased capacity for energy harvest. Nature *444*, 1027–1031.

Uusitalo, U., Liu, X., Yang, J., Aronsson, C.A., Hummel, S., Butterworth, M., Lernmark, Å., Rewers, M., Hagopian, W., She, J.X., et al.; TEDDY Study Group (2016). Association of Early Exposure of Probiotics and Islet Autoimmunity in the TEDDY Study. JAMA Pediatr. *170*, 20–28.

van Nood, E., Vrieze, A., Nieuwdorp, M., Fuentes, S., Zoetendal, E.G., de Vos, W.M., Visser, C.E., Kuijper, E.J., Bartelsman, J.F.W.M., Tijssen, J.G.P., et al. (2013). Duodenal infusion of donor feces for recurrent *Clostridium difficile*. N. Engl. J. Med. *368*, 407–415.

Vangay, P., Johnson, A.J., Ward, T.L., Kashyap, P.C., Culhane-Pera, K.A., and Knights Correspondence, D. (2018). US Immigration Westernizes the Human Gut Microbiome. Cell *175*, 962–972.

Villarino, N.F., LeCleir, G.R., Denny, J.E., Dearth, S.P., Harding, C.L., Sloan, S.S., Gribble, J.L., Campagna, S.R., Wilhelm, S.W., and Schmidt, N.W. (2016). Composition of the gut microbiota modulates the severity of malaria. Proc. Natl. Acad. Sci. USA *113*, 2235–2240.

Waki, N., Kuwabara, Y., Yoshikawa, Y., Suganuma, H., Koide, H., Oku, N., and Ohashi, N. (2017). Amelioration of Citrobacter rodentium proliferation in early stage of infection in mice by pretreatment with Lactobacillus brevis KB290 and verification using in vivo bioluminescence imaging. FEMS Microbiol. Lett. *364*, 364.

Walter, J., Armet, A.M., Finlay, B.B., and Shanahan, F. (2020). Establishing or Exaggerating Causality for the Gut Microbiome: Lessons from Human Microbiota-Associated Rodents. Cell *180*, 221–232.

Washburne, A.D., Silverman, J.D., Leff, J.W., Bennett, D.J., Darcy, J.L., Mukherjee, S., Fierer, N., and David, L.A. (2017). Phylogenetic factorization of compositional data yields lineage-level associations in microbiome datasets. PeerJ *5*, e2969.

Willing, B.P., Vacharaksa, A., Croxen, M., Thanachayanont, T., and Finlay, B.B. (2011). Altering host resistance to infections through microbial transplantation. PLoS ONE *6*, e26988.

Wu, H.J., and Wu, E. (2012). The role of gut microbiota in immune homeostasis and autoimmunity. Gut Microbes *3*, 4–14.

Yang, Y., Torchinsky, M.B., Gobert, M., Xiong, H., Xu, M., Linehan, J.L., Alonzo, F., Ng, C., Chen, A., Lin, X., et al. (2014). Focused specificity of intestinal TH17 cells towards commensal bacterial antigens. Nature *510*, 152–156.

Yatsunenko, T., Rey, F.E., Manary, M.J., Trehan, I., Dominguez-Bello, M.G., Contreras, M., Magris, M., Hidalgo, G., Baldassano, R.N., Anokhin, A.P., et al. (2012). Human gut microbiome viewed across age and geography. Nature *486*, 222–227.

Zambrano-Zaragoza, J.F., Romo-Martínez, E.J., Durán-Avelar, M.D.J., García-Magallanes, N., and Vibanco-Pérez, N. (2014). Th17 cells in autoimmune and infectious diseases. Int. J. Inflam. *2014*, 651503.

Zhang, T., Abel, S., Abel Zur Wiesch, P., Sasabe, J., Davis, B.M., Higgins, D.E., and Waldor, M.K. (2017). Deciphering the landscape of host barriers to *Listeria monocytogenes* infection. Proc. Natl. Acad. Sci. USA *114*, 6334–6339.

Zhang, P.P., Li, L.L., Han, X., Li, Q.W., Zhang, X.H., Liu, J.J., and Wang, Y. (2020). Fecal microbiota transplantation improves metabolism and gut microbiome composition in db/db mice. Acta Pharmacol. Sin. *41*, 678–685.

Zheng, P., Zeng, B., Zhou, C., Liu, M., Fang, Z., Xu, X., Zeng, L., Chen, J., Fan, S., Du, X., et al. (2016). Gut microbiome remodeling induces depressive-like behaviors through a pathway mediated by the host's metabolism. Mol. Psychiatry *21*, 786–796.

Zheng, P., Zeng, B., Liu, M., Chen, J., Pan, J., Han, Y., Liu, Y., Cheng, K., Zhou, C., Wang, H., et al. (2019). The gut microbiome from patients with schizophrenia modulates the glutamate-glutamine-GABA cycle and schizophrenia-relevant behaviors in mice. Sci. Adv. *5*, eaau8317.

Zhong, H., Ren, H., Lu, Y., Fang, C., Hou, G., Yang, Z., Chen, B., Yang, F., Zhao, Y., Shi, Z., et al. (2019). Distinct gut metagenomics and metaproteomics signatures in prediabetics and treatment-naïve type 2 diabetics. EBioMedicine *47*, 373–383.

Zopf, Y., Rabe, C., Neubert, A., Gassmann, K.G., Rascher, W., Hahn, E.G., Brune, K., and Dormann, H. (2008). Women encounter ADRs more often than do men. Eur. J. Clin. Pharmacol. *64*, 999–1004.

CellPress
OPEN ACCESS

STAR★METHODS

KEY RESOURCES TABLE

REAGENT or RESOURCE	SOURCE	IDENTIFIER
Biological samples		
US human donor fecal samples	Cornell University	N/A
Guatemalan human donor fecal samples	CeSSIAM	N/A
Fijian human donor fecal samples	FijiCOMP	N/A
Antibodies		
Rabbit anti-ki-67	Thermo Fisher	RRID: AB_2341197
Mouse anti-E-cadherin	BD Biosciences	RRID: AB_397581
Goat anti-mouse IgG secondary antibody Alexa Fluor 488	Thermo Fisher	RRID: AB_2534069
Goat anti-rabbit Rhodamine Red-x	Thermo Fisher	RRID: AB_2556551
Bacterial strains		
Citrobacter rodentium	A gift from Valerie Crepin, Imperial College London	ICC 180
Listeria monocytogenes	A gift from Brian Rudd, Cornell University	gB
Chemicals, peptides, and recombinant proteins		
Brain heart infusion broth	VWR	Cat#89405-138
LB Broth	VWR	Cat#97064-110
Antigen unmasking solution, citrate-based	Vector Laboratories	Cat#H-3300-250
DAPI	Sigma-Aldrich	Cat#D9542
Fetal bovine serum	VWR	Cat#89510
Penicillin-streptomycin	Sigma	Cat#P4333
RPMI cell culture medium	Sigma-Aldrich	Cat#R8758
AMPure XP beads	Beckman Coulter	Cat#A63880
Critical commercial assays		
Mouse TNF-alpha DuoSet ELISA	R&D Systems	DY410
Mouse IFN-gamma DuoSet ELISA	R&D Systems	DY485
Mouse IL-10 DuoSet ELISA	R&D Systems	DY417
Mouse IL-17 DuoSet ELISA	R&D Systems	DY421
Mouse IL-22 DuoSet ELISA	R&D Systems	DY582
Mouse Lipocalin-2 DuoSet ELISA	R&D Systems	DY1851
Mouse S100A8/S100A9 Heterodimer DuoSet ELISA	R&D Systems	DY582
Micro BCA protein assay kit	Thermo Fisher	Cat#23235
DNeasy PowerSoil HTP kit	QIAGEN	Cat#12955
Platinum Hot Start PCR Master Mix	Thermo Fisher	Cat#130000014
Deposited data		
V4 16S rRNA genomic sequences	This study	BioProject ID PRJNA694000
Experimental models: Mouse strains		
Germ-free C57BL/6NTac	Taconic Biosciences	Black 6 (B6NTac), B6
Germ-free C57BL/6	National Gnotobiotic Rodent Resource Center	C57BL/6
Oligonucleotides		
16S rRNA-Forward Primer 515F GTGYCAGCMGCCGCGGTAA	(Parada et al., 2016)	NA

(*Continued on next page*)

Continued		
REAGENT or RESOURCE	SOURCE	IDENTIFIER
16S rRNA-Reverse Primer 806R GGACTACNVGGGTWTCTAAT	(Apprill et al., 2015)	NA
Software and algorithms		
PRISM 9	GraphPad	https://www.graphpad.com/scientific-software/prism/
QIIME2	(Bolyen et al., 2019)	https://qiime2.org/
PhyloFactor	(Washburne et al., 2017)	https://github.com/reptalex/phylofactor
PhyloSeq	(Callahan et al., 2016)	https://joey711.github.io/phyloseq/
DADA2	(McMurdie and Holmes, 2013)	https://benjjneb.github.io/dada2/
BioRender	BioRender	https://biorender.com/
Other		
gentleMACS dissociator	Miltenyi Biotec	Cat#130-093-235
Coy vinyl anaerobic chamber	COY Lab Products	Cat#032714

RESOURCE AVAILABILITY

Lead contact

Further information and requests for resources and reagents should be directed to and will be fulfilled by the Lead Contact, Dr. Ilana Brito (ibrito@cornell.edu).

Materials availability

This study did not generate new unique reagents.

Data and code availability

- V4-16S rRNA DNA sequences are available in raw format at the NCBI's BioProject database (BioProject: PRJNA694000; https://www.ncbi.nlm.nih.gov/bioproject/694000). Relevant metadata can be found in Table S5.
- This paper does not report original code.
- Any additional information required to reanalyze the data reported in this paper is available from the lead contact upon request.

EXPERIMENTAL MODEL AND SUBJECT DETAILS

Human stool donors

Human donors were recruited in a small college town in the United States (Ithaca, NY), the third largest urban center in Guatemala (Quetzaltenango), and three agrarian villages in the Fiji Islands (Vanua Levu). Donors were required to be citizens of the respective country. Donors were recruited country between the ages of 23 and 43 years old with equal representation of subjects who identified as male and female (Table 1), Each participant provided fecal samples that were frozen within 30 minutes of collection at −20°C for a maximum of 1 hour and then moved to storage at −80°C. The samples were shipped and permanently stored at −80°C. Informed consent was obtained from all participants in accordance to the protocols approved by the Institutional Review Boards at Cornell University, Columbia University, the Massachusetts Institute of Technology, and the Broad Institute, the Human Subjects Committee at CeSSIAM in Guatemala, and the Research Ethics Review Committees at the Fiji National University and the Ministry of Health in the Fiji Islands for the Fiji Community Microbiome Project (Brito et al., 2016).

Mouse strain and husbandry

Ethical considerations

This study conformed to the National Institutes of Health guidelines on the care and use of laboratory animals. Mouse studies were performed at Cornell University (Protocol ID #2016-0088) following protocols approved by the Cornell Institutional Animal Care and Use Committee.

Mouse lines

Female germ-free C57BL/6 mice at 4-6 weeks of age were obtained from either Taconic Biosciences (*C. rodentium* experiments) or the National Gnotobiotic Rodent Resource Center at the University of North Carolina at Chapel Hill (*L. monocytogenes* experiments). Within 24 hours of arrival, the gnotobiotic mice were transferred from germ-free shipping containers to sterile filter-top cages, and orally gavaged with a human stool slurry. Mice were maintained in barrier facilities with *ad libitum* access to autoclaved water and rodent chow (autoclavable Teklad global 14% protein rodent maintenance diet #2014-S; Envigo) and water. To avoid cage effects

on the microbiota, mice were initially housed in pairs that received stool from the same human donor and were split into individual cages upon infection of one mouse per donor. Every week, food intake and animal weight were recorded, and mice were placed in clean cages with freshly autoclaved chow and water. Mice were handled under inside a biosafety cabinet with frequent glove changes and disinfection between mice during stool collection and monitoring of body weight. Stool was collected weekly throughout the course of all experiments.

Bacterial strains

The *Citrobacter rodentium* ICC800 (Crepin et al., 2016) and *Listeria monocytogenes* gB (Rudd et al., 2011) strains were obtained, respectively, from Professor Valerie Crepin (Imperial College) and Professo Brian Rudd (Cornell University). The strains were grown aerobically at 37°C in LB media (*C. rodentium*; VWR) or Brain Heart Infusion Broth (*L. monocytogenes*; VWR).

METHOD DETAILS

Preparation of human stool slurries

Frozen human feces (~100mg) were resuspended in a coy anaerobic chamber (COY Lab Products) in 1mL of reduced phosphate buffered saline (PBS) containing 0.05% L-cysteine-HCl (VWR). The fecal material sat in PBS for 15 minutes to soften followed by vortexing for 10 minutes. To separate the suspended bacteria from fibrous material, the slurries were centrifuged at 1000 g for 5 minutes. The mice were randomized into country and donor groups, and gavaged orally with 10 μL/g body weight of the corresponding slurry. Mice were monitored daily following microbiota transfer.

C. rodentium infection model

Mice were infected with *C. rodentium* ICC800 two weeks after the transfer of human-associated microbiota following protocols for this infection model outlined by Crepin et al. (Crepin et al., 2016). On the day prior to infection, a 15mL liquid culture of LB was inoculated from frozen *C. rodentium* glycerol stocks. The next day, the culture was centrifuged at 3,000xg at 4°C for 10 minutes and the pellet was resuspended in sterile PBS to obtain a final concentration of 1×10^9 CFUs/mL. For each pair of mice inoculated with stool from the same donor, one mouse per donor was inoculated with 200 μL of the *C. rodentium* suspension and the other was inoculated with 200 μL of PBS. At that point, mice were individually caged for the rest of the experiment. Mice were monitored for recovery 4-6hr after the procedure. On the day after the infection and every 2 days after, mice were weighted, and stool pellets were collected and frozen. Mice were euthanized through decapitation 14 days after inoculation with *C. rodentium* (4 weeks after arrival and gavage with human stool slurries).

Monitoring C. rodentium shedding

Stool pellets were collected on the first day after inoculation and every two days after. Samples were weighed and resuspended at a ratio of 0.1g/ml of PBS. Twenty minutes later, the stool was vortexed, and the mixture was centrifuged for 3 s at 2,500 g to separate suspended bacteria from filamentous material. Serial dilutions were prepared in PBS; 25 μL of each dilution was plated in triplicate onto LB agar containing kanamycin (50 μg/mL; VWR) (Crepin et al., 2016). CFUs were enumerated 24 hours later after incubation at 37°C.

Listeria Monocytogenes Infection Model

Mice were inoculated with *L. monocytogenes* gB 2 or 4 weeks after transfer of human-associated microbiota. Each of these experiments was conducted using stool samples from 5 unique donors per country for a total of the same 10 donors included in the *C. rodentium* experiments. Two days prior to inoculation, frozen stocks of *L. monocytogenes* were streaked onto a BHI plate and incubated at 37°C. The next day, an individual colony was picked from this plate, placed into 3mL of BHI media containing streptomycin, and incubated at 37°C overnight. The starter culture was diluted 1:10 in fresh BHI media and cultured at 37°C for 2.5-3 hours. Once the culture reached an OD_{600} between 0.4-0.7, the bacteria was centrifuged at 2,500 g for 10 mins and the pellet was resuspended in PBS to a final concentration of 1×10^5 CFU/(Smith et al., 2018; Zhang et al., 2017) One mouse per donor was inoculated with 100 μL of the *L.monocytogenes* solution and another with 100 μL of PBS through an intraocular injection. At that point, mice were individually caged for the rest of the experiment. Three days post-infection, mice were euthanized through decapitation.

Measurement of L. monocytogenes burden in the liver and spleen

Three days after inoculation with *L. monocytogenes,* the liver and spleen were collected, weighed, and placed in sterile-filtered 0.02% NP-40 (Sigma-Aldrich) in distilled deionized water. Organs were homogenized using the protein_01 program in a gentleMACS Tissue Dissociator (Milteny Biotec). Serial dilutions of these homogenates were prepared in PBS; 25 μL of each dilution was plated in triplicate on BHI plates supplemented with streptomycin and incubated at 37°C overnight (Rudd et al., 2011). Pathogen burden in the liver and spleen was then calculated after counting the *L. monocytogenes* CFUs on these plates.

Cohousing experiment

First, we identified the 5 most and 5 least susceptible mice to the *C. rodentium* infection, which happened to be those originally gavaged with stool from Fijian and Guatemalan donors, respectively. These mice were identified by comparing the area under the curve

for *C. rodentium* shedding in the stool of each individual mice in the original experiment (Table S3). Frozen cecal contents from these donor mice was resuspended in 1ml of sterile PBS and processed as described for the human stool prior to oral gavage. Two germ-free mice per donor were orally gavaged with cecum slurry within 24 hours of arrival and housed in pairs in sterile-filter top cages. Two weeks later, one mouse per least susceptible (originally GUAT) donor was randomly assigned for cohousing with one of the mice that received a transplant from the most susceptible FIJI donors. The remaining mice (one per donor) were single housed for the remainder of the experiment. Two weeks after the beginning of cohousing, all mice were inoculated with *C. rodentium*. Colonization by this pathogen was monitored for 2 weeks and the mice were euthanized 14 days after inoculation (6 weeks after arrival).

Histological and immunofluorescent characterization of explanted colons

Upon euthanasia, 0.5cm of the terminal colon was collected and cut open longitudinally. Stool was removed from the colon by quickly flushing with cold PBS using a feeding needle (Braintree Scientific Inc.). Tissue sections were fixed in formalin for at least 48 hours. The samples were then sent to the Animal Health Diagnostic Center at the Cornell University College of Veterinary Medicine for paraffin embedding, sectioning, and H&E staining. Colorimetric images of the histological stain were collected on an Olympus upright BX-50 microscope at the Cornell Institute of Biotechnology's Imaging Facility.

Prior to immunofluorescent staining, tissue sections were deparaffinized and antigen retrieval was performed for 30 minutes in citric acid buffer (pH 6.0; Vector Laboratories) at 90°C. Tissue sections were permeabilized with 0.05% Triton X-100. Autofluorescence by the tissue was quenched by incubating with 10mg/ml sodium borohydride (Sigma-Aldrich) in PBS for 30 mins at 4°C, followed by washing in PBS and blocking with 10% goat serum for 1 hour at room temperature. This was followed by staining with monoclonal antibodies against E-cadherin (1:200; BD Biosciences, RRID: AB_397581) and ki-67 (1:100, Thermo Fischer, RRID: AB_2341197) diluted in 1% goat serum in PBS overnight at 4°C. After rinsing with PBS, secondary antibodies (Thermo Fisher, RRID: AB_2534069 and AB_2556551) were applied diluted 1:500 in 1% goat serum in PBS. Finally, cell nuclei were stained with DAPI (1:500) for 10 minutes at room temperature and coverslips were placed to image the tissues. Fluorescent images were obtained on an inverted Leica DMi8 microscope.

Quantification of LCN-2 and calprotectin in mouse stool

To quantify lipocalin-2 and calprotectin levels in the stool, we followed the protocol described by Chassaing et al. (Chassaing et al., 2012). Briefly, stool pellets were weighed and reconstituted in PBS containing 0.1% Tween 20 (100mg stool per mL) followed by 10mins of vortexing. Stool sampels were then centrifuged at 10,000xg for 10 mins and the supernatant was collected and frozen at −20°C. LCN-2 and calprotectin levels were then quantified by ELISA (R&D Systems).

Quantification of inflammatory cytokine production *ex vivo*

Two small (1cm) sections of the colon were removed per mice after euthanasia and cut open longitudinally. The tissue was rinsed thrice with sterile PBS and stored at 4°C for transport. Upon arrival at the cell culture laboratory, the tissues were transferred into 500 μL of RPMI medium (Sigma Aldrich) supplemented with 10% fetal bovine serum (VWR) and 1% penicillin-streptomycin (Sigma) and incubated at 37°C for 24 hours in a humidified incubator. On the next day, the supernatants were collected and frozen at −20°C. The concentration of secreted inflammatory cytokines (TNF-α, IFN-γ, IL-10, IL-17, and IL-22) present in these *ex vivo* colonic cultures was quantified using by ELISA (R&D Systems). Cytokine concentrations were normalized to the total amount of protein in the corresponding tissue section measured using a Micro BCA protein assay kit (Thermo Fisher) after tissue lysis.

16S rRNA gene sequencing

Microbial genomic DNA was extracted from frozen human stool slurries and frozen mouse fecal pellets using QIAGEN DNeasy PowerSoil kits following the manufacturer's instructions. The extracted DNA was cleaned using AMPure XP beads (Beckman Coulter). Following standard Earth Microbiome Project protocols (Caporaso et al., 2011), the V4 region of the 16S rRNA gene was amplified in triplicate using 515F (Parada et al., 2016) and 806R (Apprill et al., 2015) primers (barcodes 515rcbc0 to 515rcbc287), and the Platinum Hot Start PCR Master Mix (Thermo Fisher). PCR products were cleaned using AMPure XP beads and pooled for each sample. The amplicon pools were then quantified with Quant-iT PicoGreen dsDNA Reagent (Invitrogen), and 100ng of amplicons per sample were pooled prior to submission for sequencing. Paired-end sequencing (2x250bp) was performed on the Illumina MiSeq platform at the Cornell Institute of Biotechnology.

QUANTIFICATION AND STATISTICAL ANALYSIS

Analysis of 16S rRNA gene sequencing

16S rRNA gene sequences were imported into the Quantitative Insights into Microbial Ecology (QIIME2; https://qiime2.org/) (Bolyen et al., 2019; Caporaso et al., 2010) pipeline for analysis. First, we used DADA2 (Callahan et al., 2016) for quality control to remove chimeric sequences, retain unique sequence variants, and trim forward and reverse reads to remove poor-quality bases. Taxonomies were assigned using QIIME2's Naive Bayes classifier trained with the SILVA rRNA database (https://www.arb-silva.de/). Prior to diversity analyses, the feature table was rarefied to 10,000 sequences per sample. We then used the 'core-metrics-phylogenetic' function to compute alpha and beta diversity metrics. The UniFrac distance matrices were then exported to R v 4.0.0 to generate principal coordinate analysis plots.

CellPress
OPEN ACCESS

To analyze the transfer of the ASVs found in the microbiomes after microbiota transplantation, ASVs that were present in fewer than 10% of samples of all three countries were removed prior to any statistical analyses, resulting in the identification of a total of 367 ASVs. We compared the presence of ASVs that were originally found in the corresponding donor, in other donors of the same country, in donors of a different country, and only found in the mice following methods reported by Fouladi et al. (Fouladi et al., 2020). Briefly, we compared the presence of ASVs between recipient mice, the corresponding human donor, and donors of the same or different countries using paired and unpaired analyses. For each mouse, we quantified ASVs that were detected only in the mouse, shared with the corresponding human donor, shared with human donors (other than the original donor) of multiple countries, or shared with human donors (other than the original donor) of a single country. The relative abundances of the ASVs belonging to each of these categories were also calculated.

PhyloFactor (choice = ‘F’, nfactor = 30), a method for identifying phylogenetic edges along which putative functional ecological traits may have arisen, was used to identify the phylogenetic factors driving *C. rodentium* colonization and bacterial shedding (Washburne et al., 2017). To analyze the transfer of microbiota from resistant to susceptible mice after cohousing, without excluding any ASVs, we compared the presence of SVs that were originally found in the mice prior to cohousing and transferred from their cohousing partners. The transfer of an ASV between cohoused mice was determined only if the ASV was detected in the recipient mouse after cohousing but not prior to it,

Human donor selection and comparison to their respective populations

To identify suitable donors for these experiments, we first selected human donors from a Guatemalan cohort recruited for this experiment and a subset of thirty-six donors from the Fiji Community Microbiome Project (Brito et al., 2016). These donors were selected taking into account age- (within 5 years and gender-matching criteria, and taxonomic composition of their microbiomes (Figure S1D). Age- and gender-matched human donors in the United States were also recruited for this experiment. In order to evaluate the suitability of these donors, we obtained additional publicly available data from another cohort in the United States (Yatsunenko et al., 2012). The sample names for the downloaded data can be found in Figure S1D. Alpha and beta diversity metrics were calculated to compare donors and their respective cohorts within and across countries. Finally, ANCOM (Mandal et al., 2015) was employed to identify differentially abundant features between donors and their respective population for all countries.

Statistical analysis

We used GraphPad Prism v9 to perform statistical analysis for all data excluding that obtained from 16S rRNA gene sequencing. Experimental groups were compared using two-way ANOVA followed by Tukey’s multiple comparisons test. Whenever time-series data was included, repeated-measured ANOVA was used instead. The specific statistical tests used and significance thresholds are indicated in figure legends.

Cell Reports, Volume *36*

Supplemental information

Geographic differences in gut microbiota composition impact susceptibility to enteric infection

Ana Maria Porras, Qiaojuan Shi, Hao Zhou, Rowan Callahan, Gabriella Montenegro-Bethancourt, Noel Solomons, and Ilana Lauren Brito

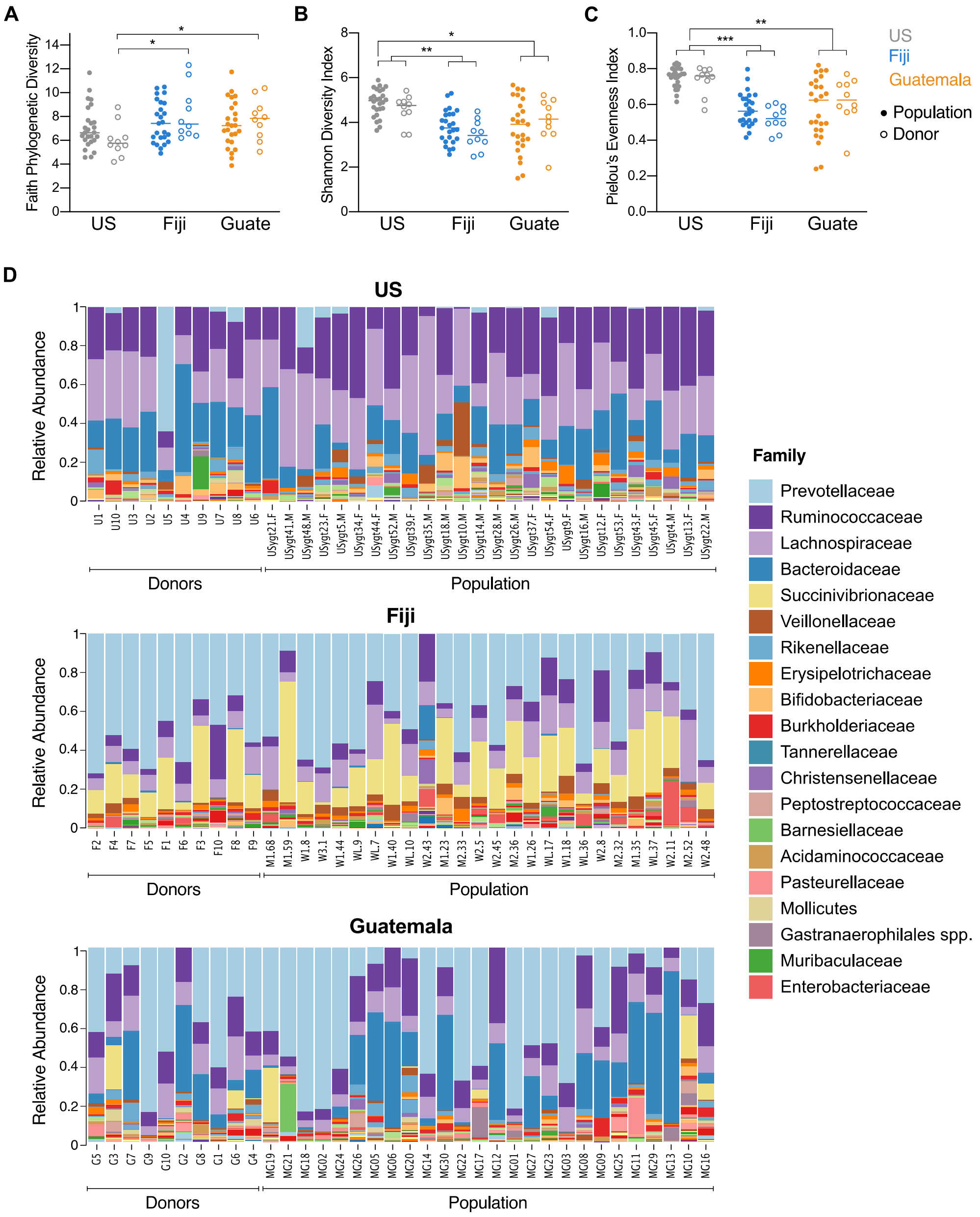

Supplementary Figure 1. The microbiomes of selected human donors are representative of those of their corresponding populations. Alpha diversity of the gut microbiomes of both donors and subjects from their populations were analyzed through (A) the Faith phylogenetic diversity index, (B) the Shannon diversity index and (C) Pielou's evenness index. (D) Abundance of bacteria genera in donors and their respective populations. Each bar represents an individual. Data are presented as mean values ± SD, with *p<0.05, **p<0.01, and ***p<0.001 for comparisons shown (one-way ANOVA followed by Tukey's multiple comparison test). Related to Figure 1.

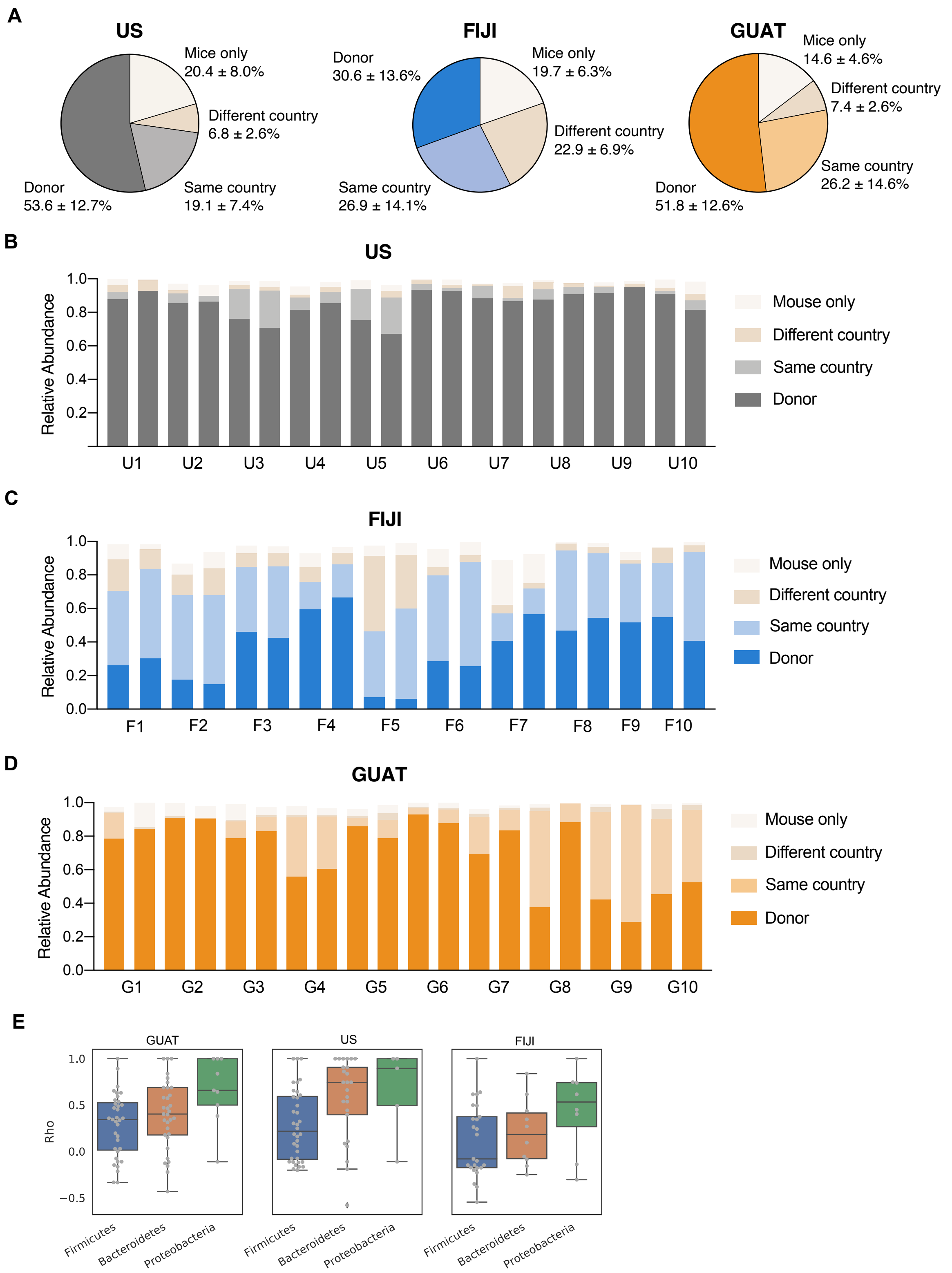

Supplementary Figure 2. Human-associated microbes successfully colonize germ-free mice. (A) Proportion of the SVs found in the microbiomes of the mice 14 days after microbiota transplantation that were originally found in the corresponding donor, in other donors of the same country, in donors of a different country, and only found in the

mice. We also calculated the relative abundances of the SVs found in the microbiomes of the (B) US, (C) FIJI, and (D) GUAT mice 14 days after microbiota transplantation that were originally found in: the corresponding donor, in other donors of the same country, in donors of a different country, or only found in the mice. (E) Boxplots showing colonization efficiency of ASVs transferred from the human microbiota to the mice at the phylum level 2 weeks after colonization. Transfer efficiency is represented by the Spearman correlation coefficients (rho). Related to Figure 1.

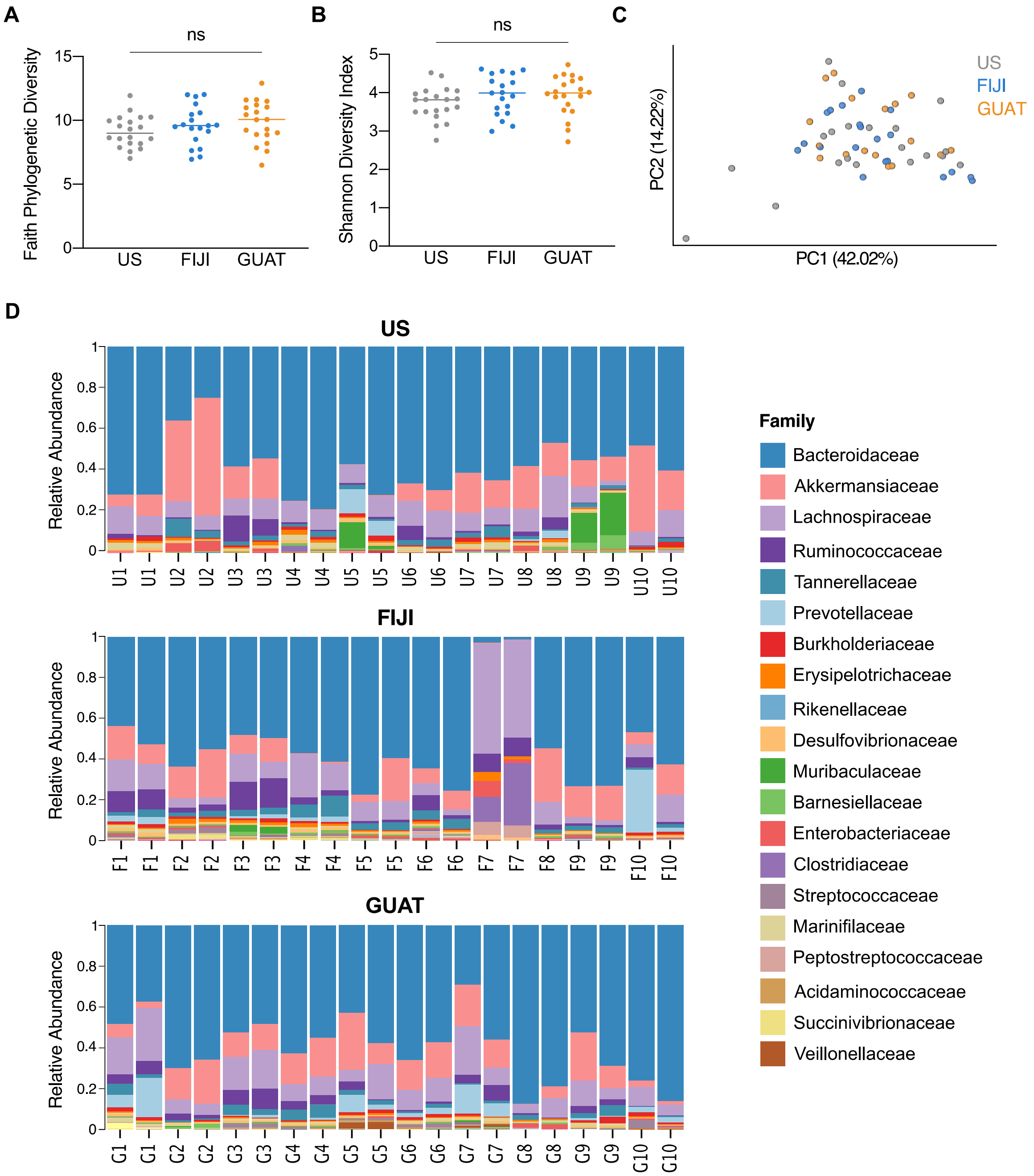

Supplementary Figure 3. Composition of the US, FIJI, and GUAT mouse microbiomes 14 days post-microbiota transplantation. Alpha diversity of the mouse gut microbiomes were analyzed through (A) the Faith phylogenetic diversity index and (B) the Shannon diversity index. (C) PCoA of weighted UniFrac distances for the gut microbiomes of the humanized mice 14 days after colonization with donor-associated microbiota. Horizontal bar represents the mean. No statistically significant differences were observed between countries (ns = not significant, one-way ANOVA followed by Tukey's multiple comparison test). (D) Taxa bar plots for all mice grouped at the family level. Each bar represents an individual mouse. Labels on the x-axis denote the original human donor. Related to Figure 1.

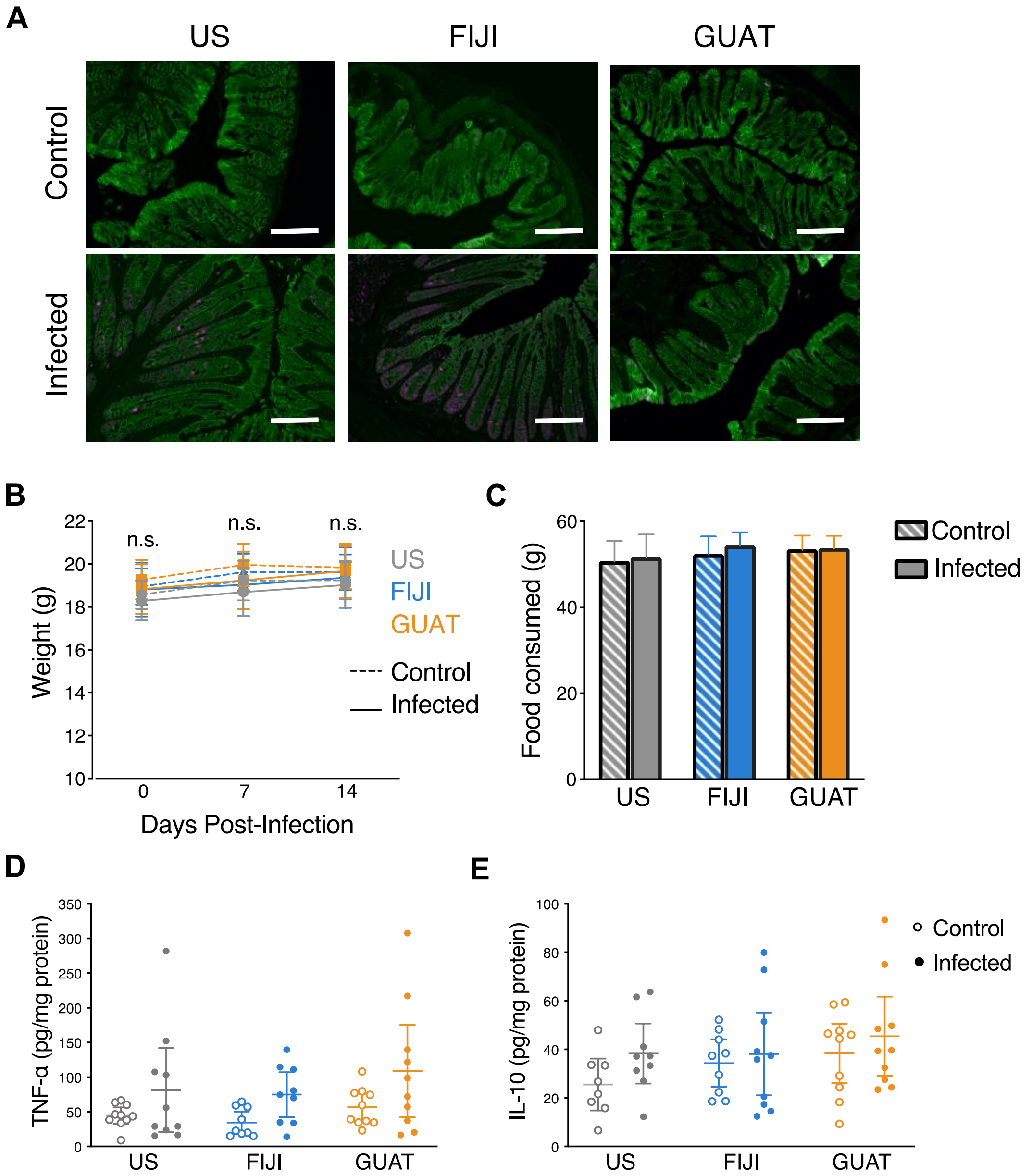

Supplementary Figure 4. Geographic differences in the microbiome lead to differences in proliferation but not in weight loss or food consumption in response to *C. rodentium* infection. (A) Representative images of Immunofluorescent staining of ki67, a marker for cell proliferation, in colon sections of control and infected mice 14 days after infection with *C. rodentium*. E-cadherin (cell junctions) is stained green; ki67 is stained magenta. Scale bar represents 100µm. (B) Mouse weight at days 0, 7, and 14 post-infection for all experimental groups. (C) Food consumption between inoculation with *C. rodentium* and 14 days post-infection. (D-E) Quantification of the production of the inflammatory cytokines (D) TNF-α, and (E) IL-10 in *ex vivo* colon culture. Data are presented as mean values ± SD, n.s. = not significant for comparisons shown (one-way ANOVA followed by Tukey's multiple comparison test). Related to Figure 1.

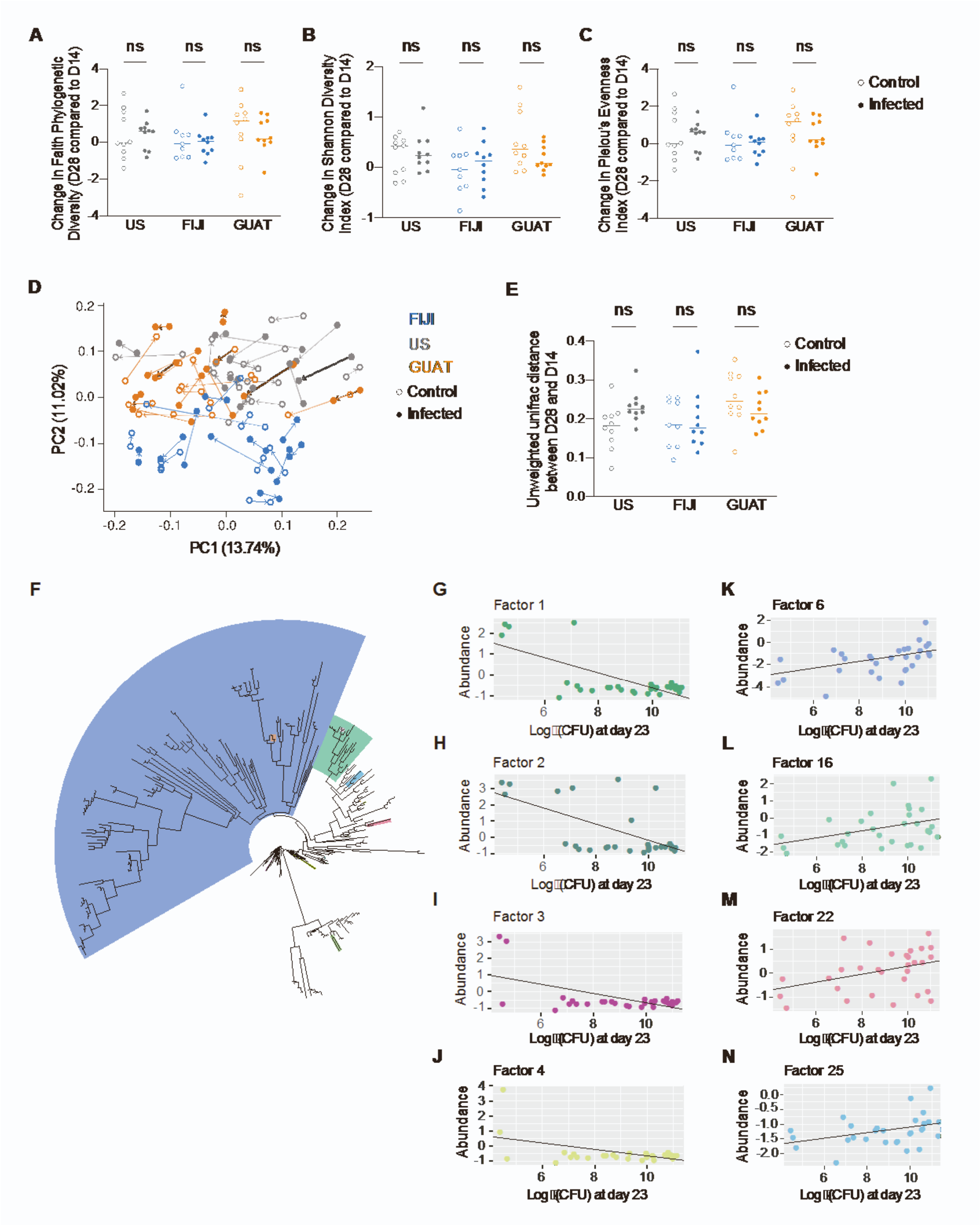

Supplementary Figure 5. Characterization of the changes in the mouse microbiomes after *C. rodentium* infection. Quantification of the changes in (A) the Faith phylogenetic diversity index, (B) the Shannon diversity index and (C) Pielou's evenness index before and after infection. (D) PCoA of unweighted UniFrac distances for the gut microbiomes of the humanized mice 14 (pre-infection) and 28 (end of the experiment) days after colonization with

donor-associated microbiota. Arrows originate at day 14 and end at day 28 for the corresponding sample. (E) Quantification of the unweighted Unifrac distance between days 28 and day 14. Horizontal lines represent the mean, ns = not significant for comparisons shown (one-way ANOVA followed by Tukey's multiple comparison test). We also attempted to identify the phylogenetic factors driving *C. rodentium* colonization and shedding using PhyloFactor. (F) Phylogenetic tree of all the ASVs identified across all microbiomes, different colors represent the biggest clades identified as important factors in the analysis. Groups that consist of a single tip are not depicted. (G-N) Linear models depicting the association between specific factors and bacterial shedding at day 23. The line represents predicted values and the dots observed abundances. Related to Figure 1.

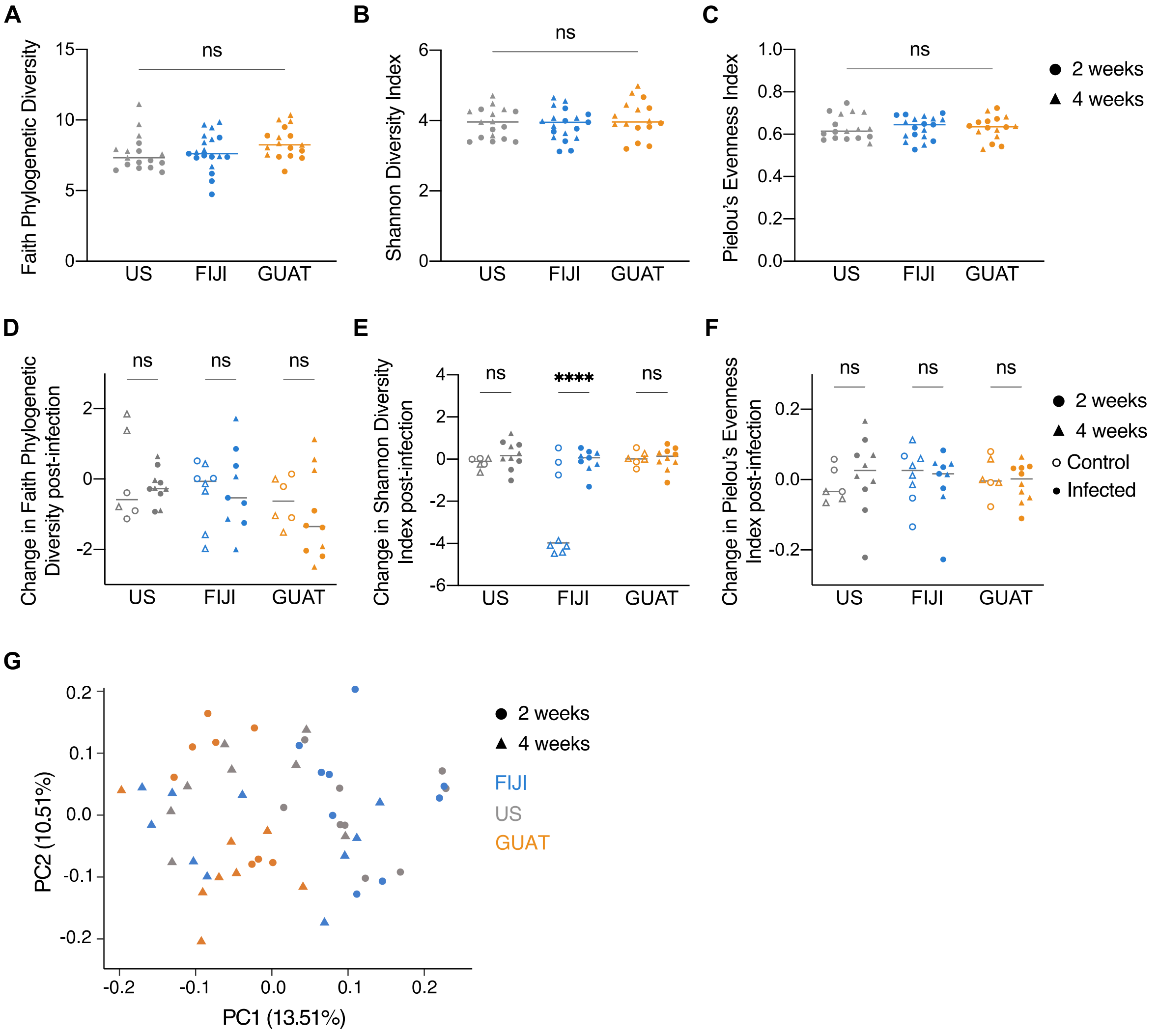

Supplementary Figure 6. Analysis of the US, FIJI, and GUAT mouse microbiomes before and after infection with *L. monocytogenes.* Alpha diversity of the mouse gut microbiomes 14 days after microbiota transplantation was analyzed through (A) the Faith phylogenetic diversity index, (B) the Shannon diversity index and (C) Pielou's evenness index. Changes in alpha diversity metrics were also quantified by comparing the microbiomes right before the infection (at days 14 for the 2-week experiment or day 28 for the 4-week experiment) and three days later. Differences in (D) the Faith phylogenetic diversity index, (E) the Shannon diversity index and (F) Pielou's evenness index were calculated. Horizontal lines represent the mean, ****$p<0.0001$, ns = not significant for comparisons shown (one-way ANOVA followed by Tukey's multiple comparison test). (G) PCoA of unweighted UniFrac distances for the gut microbiomes of the humanized mice immediately prior to infection. Related to Figure 3.

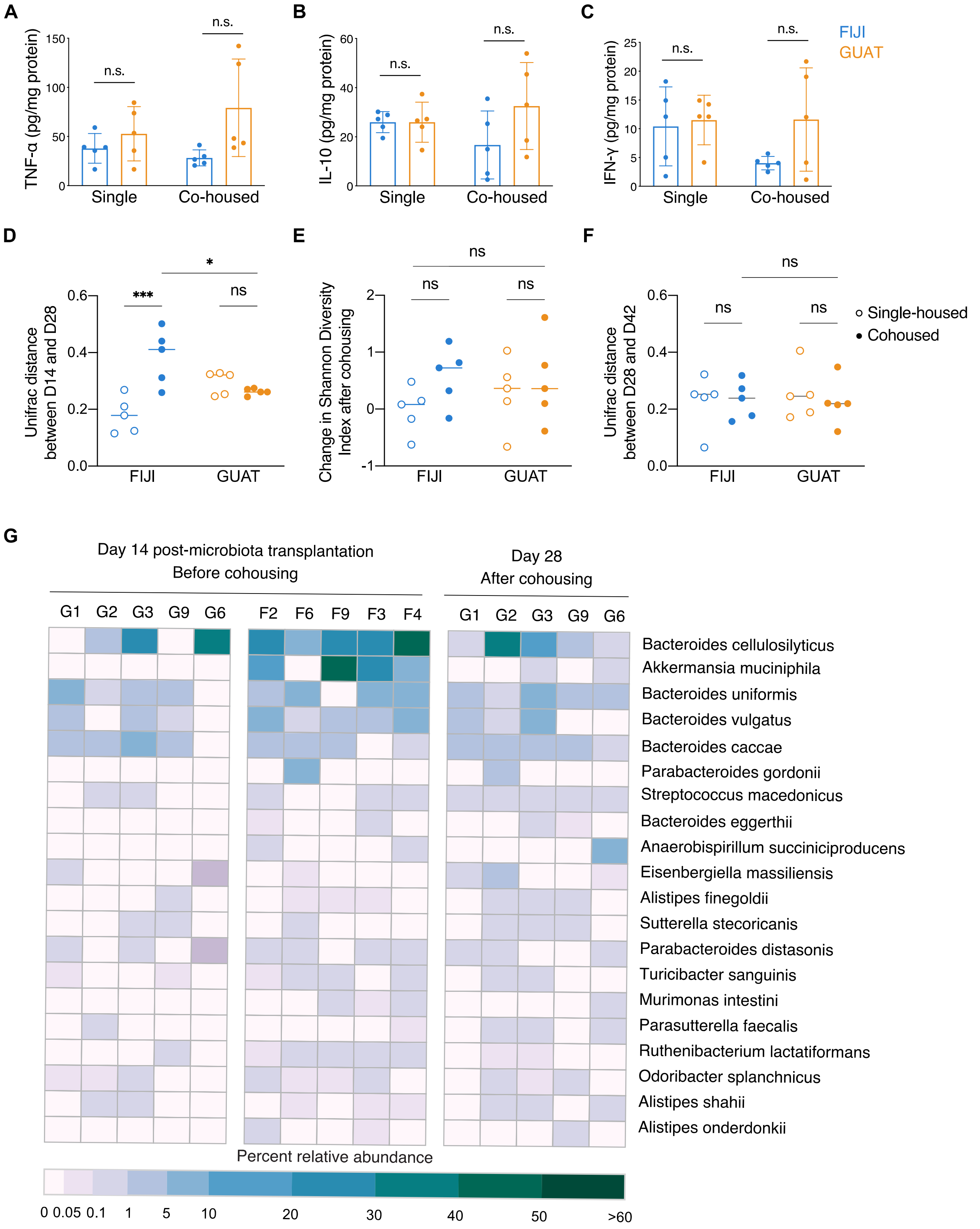

Supplementary Figure 7. Quantification of changes in inflammatory cytokine secretion and microbiota for all mice after cohousing and infection with *C. rodentium*. (A-C) Quantification of the production of the inflammatory cytokines (A) TNF-α, (B) IL-10, and (C) IFN-γ in *ex vivo* colon culture. Quantification of (D) the Unifrac distance of

each mouse's microbiome between days 14 and 28 – cohousing, (E) changes in the Shannon diversity index, between days 14 and 28 as a result of cohousing, and (F) Unifrac distance of each mouse's microbiome between (J) days 28 and 42 – infection. Horizontal lines represent the mean with standard deviation were depicted; ns = not significant, *p<0.05, ***p<0.001 for comparisons shown (one-way ANOVA followed by Tukey's multiple comparison test). Related to Figures 4 and 5.

Supplementary Table 1. Differentially abundant families between donor and population m

No differentially abundant families were identified in the Guatemalan cohort. Related to Figure 1

Percentile abundances of differentially abundant families in the US cohort										
	Donors					Population				
**Family**	**0**	**25**	**50**	**75**	**100**	**0**	**25**	**50**	**75**	**100**
Succinivibrionaceae	1	1	2	5.5	15	1	1	1	1	1
Streptococcaceae	1	1	8	11.25	32	1	18.5	27	183	652
Erysipelotrichaceae	10	16	91.5	181	518	41	194	412	950	2995
Desulfovibrionaceae	1	6.25	12	30.25	55	1	26.75	87.5	125	469
Lachnospiraceae	495	1204	2635	3554	14968	2735	5099	8014	11044	18020
Anaeroplasmataceae	1	1	1	1	4	1	1	1	1	1
Elusimicrobiaceae	1	1	1	1	3	1	1	1	1	1
Tannerellaceae	1	19.25	73	174.3	414	1	237.8	385.5	676	1383

Percentile abundances of differentially abundant families in the Fiji cohort										
	Donors					Population				
**Family**	**0**	**25**	**50**	**75**	**100**	**0**	**25**	**50**	**75**	**100**
Molicutes	1	8.5	16.5	51.25	616	1	1	1	5.75	158

Supplementary Table 3. Area under the *C. rodentium* shedding curve ranked in descending order. Related to Figure 4.

Mouse ID	Country	AUC
230	Fiji	3.686E+11
218	Fiji	2.7765E+11
220	Fiji	2.4156E+11
225	Fiji	2.2443E+11
327	Fiji	1.7446E+11
311	Fiji	1.2829E+11
222	Fiji	1.2425E+11
315	Fiji	7.1891E+10
226	US	6.3441E+10
303	Fiji	4.9811E+10
206	US	3.4548E+10
322	US	2.5877E+10
329	US	2.4714E+10
305	US	1.6616E+10
325	Guatemala	1.0298E+10
319	Guatemala	7050120000
221	US	6999058300
310	Guatemala	4575879300
318	US	3954273350
213	Guatemala	3948202000
205	US	2218280000
211	US	1843546800
309	Fiji	1505363000
214	Guatemala	784928800
313	US	725066950
229	Guatemala	713095000
204	Guatemala	362752660
324	Guatemala	108296500
330	Guatemala	15600600
212	Guatemala	3745600
313	US	713095000
